# Clustering and Alignment of Polymorphic Sequences for *HLA-DRB1* Genotyping

**DOI:** 10.1371/journal.pone.0059835

**Published:** 2013-03-28

**Authors:** Steven Ringquist, Gaia Bellone, Ying Lu, Kathryn Roeder, Massimo Trucco

**Affiliations:** 1 Department of Pediatrics, Division of Immunogenetics, Children’s Hospital of Pittsburgh of UPMC, University of Pittsburgh School of Medicine, Pittsburgh, Pennsylvania, United States of America; 2 Department of Statistics, Carnegie Mellon University, Pittsburgh, Pennsylvania, United States of America; Centro Cardiologico Monzino IRCCS, Italy

## Abstract

Located on Chromosome 6p21, classical human leukocyte antigen genes are highly polymorphic. *HLA* alleles associate with a variety of phenotypes, such as narcolepsy, autoimmunity, as well as immunologic response to infectious disease. Moreover, high resolution genotyping of these loci is critical to achieving long-term survival of allogeneic transplants. Development of methods to obtain high resolution analysis of *HLA* genotypes will lead to improved understanding of how select alleles contribute to human health and disease risk. Genomic DNAs were obtained from a cohort of n = 383 subjects recruited as part of an Ulcerative Colitis study and analyzed for *HLA-DRB1*. *HLA* genotypes were determined using sequence specific oligonucleotide probes and by next-generation sequencing using the Roche/454 GSFLX instrument. The Clustering and Alignment of Polymorphic Sequences (CAPSeq) software application was developed to analyze next-generation sequencing data. The application generates *HLA* sequence specific 6-digit genotype information from next-generation sequencing data using MUMmer to align sequences and the R package diffusionMap to classify sequences into their respective allelic groups. The incorporation of Bootstrap Aggregating, Bagging to aid in sorting of sequences into allele classes resulted in improved genotyping accuracy. Using Bagging iterations equal to 60, the genotyping results obtained using CAPSeq when compared with sequence specific oligonucleotide probe characterized 4-digit genotypes exhibited high rates of concordance, matching at 759 out of 766 (99.1%) alleles.

## Introduction

The classical human leukocyte antigen (*HLA*) loci *-A*, *-B*, and *-C* (class I) as well as *-DRB1*, *-DQB1*, and *-DPB1* (class II) are expressed on the cell surface and function as antigen-presenting proteins. *HLA* loci were first identified during studies involving transplantation of tumor cells among different strains of mice, and later shown to be the principle genetic barrier to allogeneic transplantation of cells, tissues, and organs [1]. In humans, these genes are clustered within a roughly 4 Mb region on Chromosome 6p21 [2]. The genes encoding the HLA proteins are characterized by frequent polymorphisms, occurring as often as 1 out 8 nucleotides, resulting in greater than 7,800 characterized alleles and more than 5,700 protein variants [3]. Select alleles have been implicated in pharmacogenomics [4] and human diseases, associating with immune response to infectious agents as well as autoimmunity and narcolepsy [2].

The impact of *HLA* diversity on transplant genetics, the requirement to match *HLA* alleles between unrelated donor and recipient, is that there is currently an estimated 60% chance of finding a matched donor-recipient pair when genotyping for *HLA-A*, *-B*, and *-DRB1* [5], [6]. Timely matching of donor with recipient, therefore, depends upon ongoing recruitment of volunteer donors. Currently, screening of *HLA* genotypes from increasingly large cohorts is achieved using the sequence specific oligonucleotide (SSO) typing approach. In this methodology, genomic DNA is PCR amplified and the product detected by hybridization of allele specific probes [7]. When determining 4-digit resolution (e.g., HLA-DRB1*15∶01 and denoting the protein sequence subtype) the SSO method is highly accurate and can be performed rapidly [8]. For example, the overall accuracy of SSO based genotyping of *HLA-DRB1* is estimated at roughly 99% based upon blinded analysis of 1,652 reference samples [8], however, full resolution genotyping of alleles defining substitutions occurring among synonymous codons as well as within introns and untranslated regions are not routinely achieved.

In contrast, full resolution genotyping of *HLA* loci can be achieved by next-generation sequencing based protocols [9], [10]. Since it was first developed pyrosequencing technology has advanced substantially allowing it to be used in ways that enable efficient preparation of thousands of individually prepared clones for analysis within a single sequencing run, scaling of the reaction to picoliter volumes, and increasing read lengths to several hundred nucleotides [11]. As the length of the polymorphic exons of *HLA* class I (exons 2 and 3) and class II loci (exon 2) are between 264 base pairs for *HLA-DPB1* exon 2 and 276 base pairs for exon 3 of *HLA-A*, *-B*, and *-C* the method is capable of sequence based typing of *HLA* alleles. In fact, a number of research laboratories have already reported achieving high resolution typing of *HLA* class I and class II loci using genomic DNA isolated from human cell lines as well as blood samples [9], [10], [12], [13] and from cDNA created from isolations of mRNA [14], [15]. Along with translating the methodology from the research based setting to the *HLA* genotyping laboratory there is a need to develop robust computational methods for analyzing the data [12], [16], [17].

## Materials and Methods

### Ethics Statement

All subjects were recruited for genetic studies at the University of Pittsburgh under institutional review board approved protocols [18].

### Materials

Residual DNA samples were selected from cohorts recruited as part of a study into the genetics of Ulcerative Colitis (UC). The UC cohort had been previously analyzed during a genome-wide scan of SNPs and the 4-digit *HLA-DRB1* genotypes were inferred via the imputation of SNPs approach and confirmed by SSO genotyping [18]. Individual DNA samples (n = 383), representing a subset of subjects recruited for the UC cohort, were chosen for next-generation sequencing in order to capture a wide range of alleles (Table 1). *HLA-DRB1* alleles covered by the UC cohort accounted for 36 commonly occurring alleles, including 29 out of the 31 most frequently occurring alleles observed among subjects of European ancestry [19]. The cumulative frequency of allelic variance covered for *-DRB1* was 99.5% among European populations and 84.3% among populations worldwide [19], [20].

**Table 1:** *HLA-DRB1* alleles.

HLA-DRB1	European Freq (Rank)	Worldwide Freq (Rank)	UC Cohort Freq
*01∶01	0.09149 (4)	0.04123 (8)	0.07311
*01∶02	0.01703 (13)	0.01161 (26)	0.02872
*01∶03	0.00889 (19)	0.00329 (41)	0.02480
*03∶01	0.12916 (3)	0.06760 (3)	0.10183
*04∶01	0.09111 (5)	0.02896 (13)	0.07963
*04∶02	0.00972 (17)	0.00742 (32)	0.00392
*04∶03	0.00572 (23)	0.02659 (17)	0.00392
*04∶04	0.03634 (9)	0.01795 (19)	0.02219
*04∶05	0.00368 (25)	0.04776 (6)	0.00522
*04∶07	0.00947 (18)	0.01536 (23)	0.00783
*04∶08	0.00248 (26)	0.00188 (43)	0.00261
*07∶01	0.13767 (2)	0.06986 (2)	0.10574
*08∶01	0.02363 (12)	0.00875 (29)	0.03525
*08∶02	0.00025 (34)	0.02104 (17)	0.00131
*08∶03	0.00133 (29)	0.03864 (9)	0.00261
*08∶04	0.00089 (31)	0.00500 (36)	0.00392
*08∶06	0.00006 (41)	0.00101 (47)	0.00131
*08∶10	0.00000 (NA)	0.00003 (68)	0.00131
*09∶01	0.00820 (21)	0.05450 (5)	0.01175
*10∶01	0.00826 (20)	0.01284 (24)	0.01044
*11∶01	0.05654 (7)	0.05945 (4)	0.08747
*11∶02	0.00152 (28)	0.00604 (33)	0.00131
*11∶03	0.00483 (24)	0.00227 (42)	0.00522
*11∶04	0.03189 (10)	0.01780 (20)	0.04178
*11∶14	0.00000 (NA)	0.00003 (68)	0.00131
*11∶15	0.00000 (NA)	0.00000 (NA)	0.00131
*11∶29	0.00000 (NA)	0.00002 (69)	0.00131
*12∶01	0.01468 (14)	0.02712 (16)	0.03003
*13∶01	0.06283 (6)	0.03152 (12)	0.05875
*13∶02	0.04015 (8)	0.03746 (10)	0.03655
*13∶03	0.00991 (16)	0.00784 (30)	0.01436
*13∶27	0.00000 (NA)	0.00000 (NA)	0.00131
*14∶01	0.02459 (11)	0.03218 (11)	0.02742
*15∶01	0.14441 (1)	0.07864 (1)	0.13316
*15∶02	0.00775 (22)	0.04507 (7)	0.00653
*16∶01	0.01061 (15)	0.01656 (21)	0.02480
**Study Panel Total**	**0.995**	**0.843**	

Allele frequencies and rankings are taken from Maiers et al. [19] for European *-DRB1* and from Lancaster et al. [20] for worldwide frequencies. UC cohort frequencies are determined from CAPSeq genotyping results.

### Amplicon Preparation

Forward barcoded oligonucleotide primer 5′-CGTATCGCCTCCCTCGCGCCATCAG[MID]CCGGATCCTTCGTGTCCCCACAGCAC-3′ employing a multiplex identifier region (MID) and reverse primer 5′-CTATGCGCCTTGCCAGCCCGCTCAGCCGCTGCACTGTGAAGCTCTC-3′ were used to target *HLA-DRB1* exon 2 for amplification, and were chimeras of titanium primers A and B along with *HLA-DRB* amplification primers [9]. DNA amplification was performed in 10 µl and used 40 ng genomic DNA, 400 nM oligonucleotide primers, 0.2 mM dNTP, and 1 unit FastStart High Fidelity Enzyme Blend (Roche Diagnostics). Thermal cycling was performed by incubation at 94°C for 3 minutes followed by 40 cycles at 94°C for 30 seconds, 61°C for 45 seconds, and 72°C for 1 minute. The reactions were then incubated at 72°C for 2 minutes.

### Next-Generation Sequencing

Next-generation sequencing was performed using the Roche/454 titanium assay as described previously [18]. Briefly, emulsion of PCR reagents in microreactors was prepared by mixing beads, PCR reaction mix (1X amplification mix, Amplification Primers, 0.15 U/µl Platinum Taq (Invitrogen, Carlsbad, CA)), and emulsion oil and mixing vigorously using a Tissue Lyser (Qiagen, Valencia, CA). Emulsion was distributed into a PCR plate and template amplification was carried out in a thermocycler using the following cycling conditions: Hotstart activation for 4 minutes at 94°C, 40 cycles of 94°C for 30 seconds, 58°C for 1 minute, 68°C for 90 seconds followed by 13 cycles of 94°C for 30 seconds and 58°C for 6 minutes. Sequencing primers were added to the mixture of beads and annealing buffer and annealed to the template using the following thermocycler conditions: 65°C for 5 minutes, ramp to 50°C at 0.1°C/second, hold at 50°C for 1 minute, ramp to 40°C at 0.1°C/second, hold at 40°C for 1 minute, ramp to 15°C at 0.1°C/second, hold 15°C. Packing beads, sample beads and enzyme beads were applied to the picotiter plate as per manufacturer instructions. The sequencing reaction was performed in picotiter plates loaded onto the sequencer. The Roche/454 instrument used pyrosequencing chemistry and detected the incorporation of each nucleotide in real time.

### CAPSeq Genotyping

**Figure 1:**
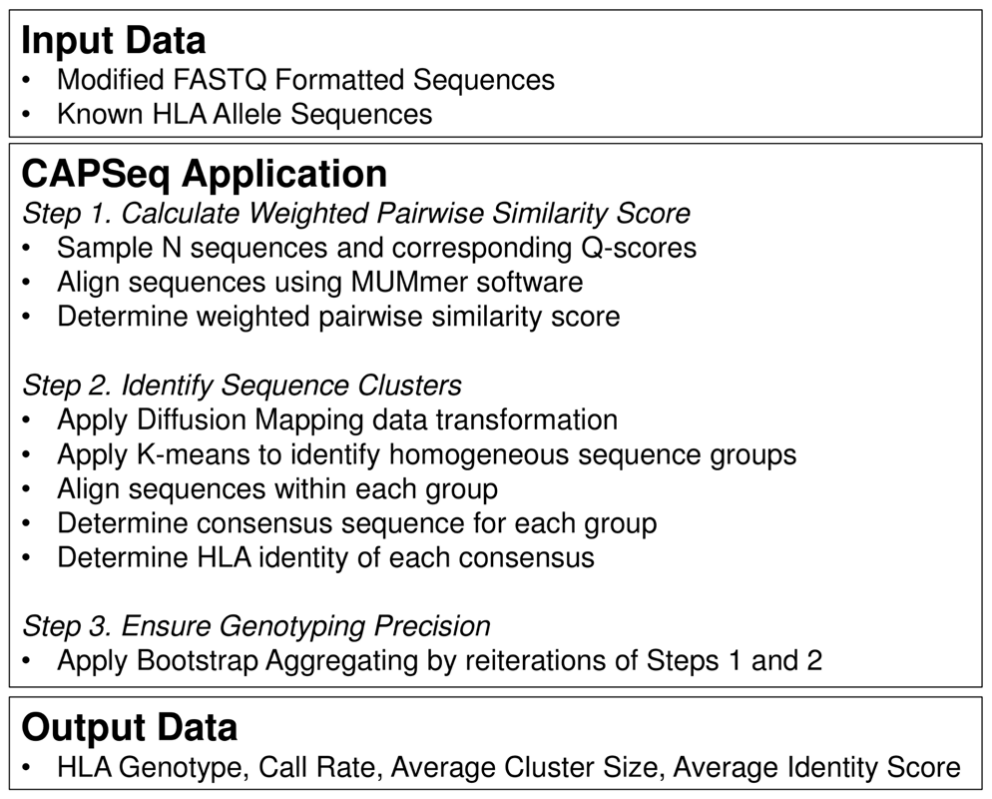
The Clustering and Alignment of Polymorphic Sequences (CAPSeq) software application illustrated as a schematic. *Input Data:* Next-generation sequence data formatted as modified FASTQ files consisting of sequences and corresponding Q-scores along with an additional input data file containing known *HLA* allele sequences. *CAPSeq Application:* The analysis software can be broken down into 3 principle steps consisting of those developed to align sequences and use corresponding Q-scores to generate a weighted pairwise similarity score (step 1) that can be analyzed via diffusion mapping, followed by K-means clustering to enable the identification of homogeneous sequence groups (step 2) followed by Bootstrap Aggregating, Bagging, of multiple analyses of the data to ensure genotyping precision (step 3). *Output Data:* The *HLA* genotyping data is provided as a tab delimited text file containing the most likely allelic match between the CAPSeq generated consensus sequences and list of known *HLA* alleles.

*HLA* genotypes were determined from sequence data using the CAPSeq software application (Figure 1). The CAPSeq application uses next-generation sequences and their corresponding Q-Scores to enable alignment followed by classification of sequences into their corresponding allelic components. The consensus sequences are then compared with known *HLA* alleles [3] and the nearest match reported as the genotype. The application is written in R and PERL and can be run using the R command source (“CAPSeq.R”). The software is compatible with Linux and Mac operating systems.

### Online Software and Sample Data

The CAPSeq software and *HLA-DRB1* sample data are available at (http://wpicr.wpic.pitt.edu/WPICCompGen/) and consist of four R Scripts CAPSeq.R, CompleteTmp.R, diffuse.R, GenoFuns1.R; the six PERL Scripts CreateReadsQscore.pl, Edit_Alignment.pl, Edit_Nucmer_SNP.pl, Format_Delta_Files.pl, Identity_Score.pl, Matrix_Check.pl; and the *HLA-DRB1* Sequence File Library_DRB_Release370_unique_exon2. Sample Data obtained from five subjects are available in the folder DRB_Example and are formatted for use with the CAPSeq application. All new data have been deposited in GenBank (SUB162897). The ReadMe file provides directions for installing and running the CAPSeq application.

## Results

### Next-generation Sequencing

Next-generation sequencing analysis of *HLA* loci *-DRB1* exon 2 was performed using the Roche/454 GSFLX instrument and resulted in 371,657 sequences from the UC cohort of 383 subjects. Sequencing assays were performed so as to obtain data from both strands. Upon analysis of read lengths for those sufficient to enable direct analysis of exon 2, that is, without the need to assemble truncated DNA sequences there were 368,610 sequences remaining, accounting for 99% of the total. After data cleaning in which sequence barcodes were used to identify subjects [21] and sequence motifs found within *HLA* exons were used to identify the location of the *-DRB1* exon start and finish boundaries there were 330,981 (89%) of the sequences remaining. Due to the complexity of the *HLA-DRB* sub-region in which amplicons in addition to those at the *HLA-DRB1* locus were generated the data were subjected to additional cleaning steps designed to minimize the occurrence of sequences containing motifs found only in *HLA-DRB* pseudogenes (e.g., *HLA-DRB6/7/8/9*) and paralogous loci (i.e., *HLA-DRB3/4/5*). After data cleaning there were 130,513 (35%) sequences remaining corresponding to a mean and standard deviation of 340±132 sequences per subject.

### HLA-DRB1 Genotyping

The Clustering and Alignment of Polymorphic Sequences (CAPSeq) software makes use of statistical analysis for calling each genotype. The CAPSeq application incorporates MUMmer [22] to align sequences and the R package diffusionMap [23], [24] to classify the sequence data into allelic groups. The genotyping process is organized into three main steps (Figure 1). The first step consists in identifying the clusters of reads generated by the different alleles. To do this, a pairwise weighted similarity score is computed as the difference between the alignment length and the number of mismatches among the aligned sequences. At this stage the information about the variable regions are incorporated via their overall sequence quality score, giving lower weight to the mismatches belonging to these regions (e.g., when Q-Scores are less than 30). Second, Diffusion Mapping [23], [24] is used to generate a data transformation that can simplify cluster structures and protect from the presence of outliers (e.g., sequence reads containing infrequent variants generated during PCR amplification). The data representing the pairwise weighted similarity score are projected into diffusion space in which the Euclidean distance between two points is small if the points are highly connected in the original feature space and large otherwise. Then the K-means algorithm is applied and the reads are clustered into homogeneous groups. During this process the consensus sequence for each sequenced cluster is established. The genotype is determined by comparing the consensus sequence with that of known *HLA* allele sequences obtained from the HLA/IMGT database [3]. A third step is performed for ensuring classification precision. To this end Bootstrap Aggregating, Bagging, is used [25]. Bagging is a machine learning technique that generates multiple versions of the same predictor (e.g., by random sampling of sequence reads), taking an “average” as the final result. In the present example it consists in sampling a subset of N = 20 reads, while performing steps one and two in an iterative fashion.

The genotyping results obtained using CAPSeq software are summarized in Table 2. Analysis was performed in quadruplicate at increasing Bagging iterations from 5 to 60 times. A greater number of iterations lead to improved identification of the *HLA-DRB1* genotypes. For example, the rate of concordance between CAPSeq and SSO determined genotypes improved with increasing Bagging from 95.7% at Bagging 5 to 99.1% when Bagging was performed 60 times. The improvement resulted from a decreased number of sensitivity errors occurring when a genotype determined as heterozygous via the SSO approach was reported as homozygous during CAPSeq analysis of the next-generation sequencing data. For instance, there was a roughly 6-fold decrease in sensitivity errors when Bagging iterations were increased with 5 persistent sensitivity errors occurring at Bagging 60 compared to 31 sensitivity errors observed at Bagging 5 (Table 2).

**Table 2:** Comparison of SSO based 4-digit *HLA-DRB1* genotyping with CAPSeq.

BaggingIterations	Concordant	Sensitivity Error	Specificity Error
5	733 (95.7%)	31 (4.0%)	2 (0.3%)
10	747 (97.5%)	17 (2.2%)	2 (0.3%)
20	752 (98.2%)	11 (1.4%)	3 (0.4%)
40	758 (99.0%)	6 (0.8%)	2 (0.3%)
60	759 (99.1%)	5 (0.7%)	2 (0.3%)

**Figure 2:**
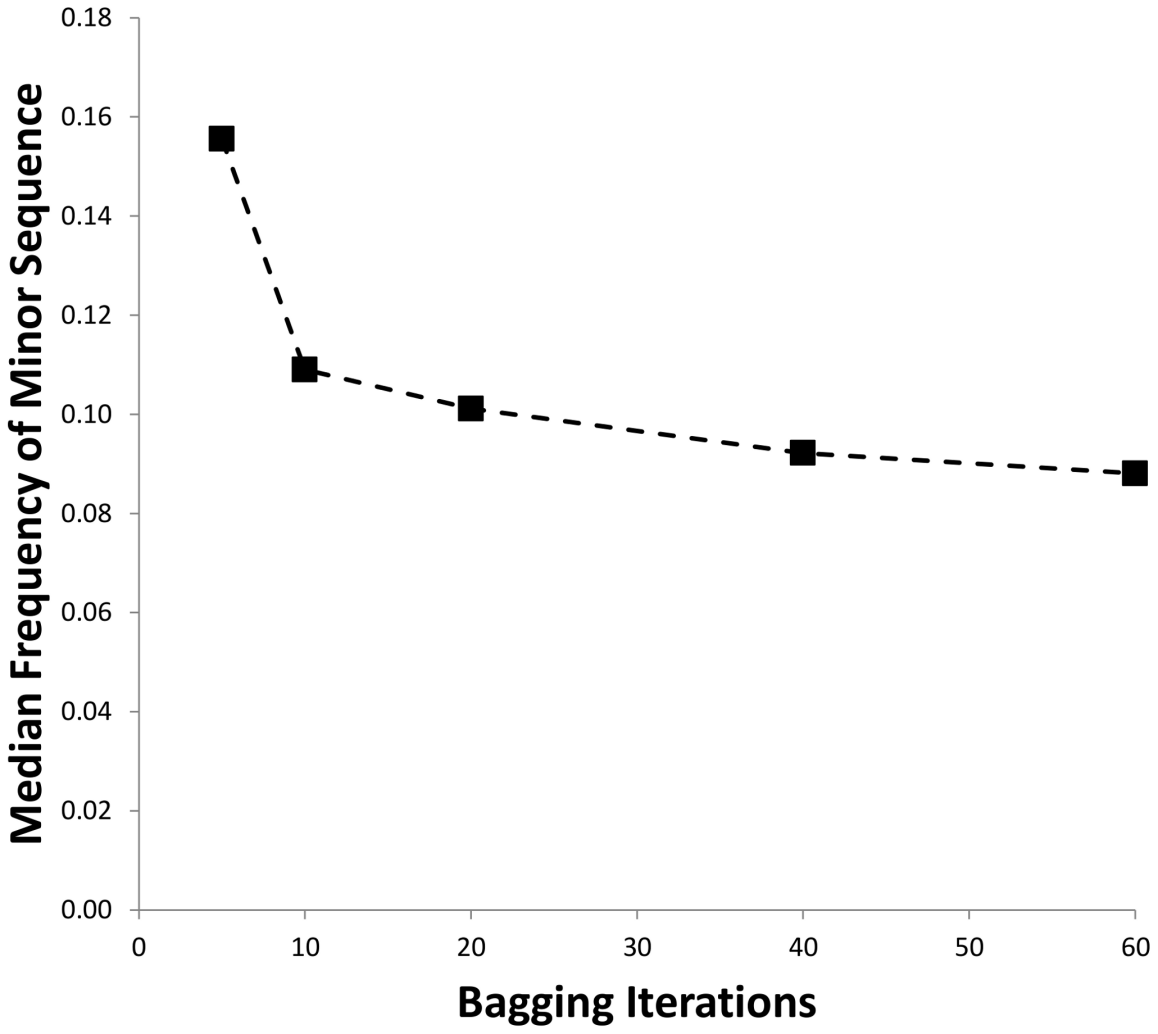
CAPSeq Bagging iterations result in improved genotyping sensitivity. Bagging iterations (x-axis) were varied from 5 to 60. The median frequency of the minor sequence that was detectable by CAPSeq (y-axis) is determined from interrogation of the raw sequencing data obtained using the Roche/454 GSFLX instrument.

Listed in Supplementary Table S1 are the complete sets of 6-digit CAPSeq genotyping results for Bagging iterations set at 60 along with the 4-digit genotyping results obtained using the SSO approach. The roughly 1% of CAPSeq called alleles that were discordant when compared with the results of SSO based genotyping are identified by bold font. The CAPSeq data also include “Call Rate” (the frequency with which each allele is detected during the Bagging iterations), “Average Cluster Size” (the average number of times each allele is observed during sampling of the sequence data), and “Average Identity Score” (how well the final CAPSeq derived consensus sequence matched the sequence of the closest known *HLA-DRB1* allele), exhibiting overall mean and standard deviation values 93±18, 52±18, and 100±0, respectively. Discordant calls were attributable to a combination of sensitivity errors occurring 0.7% (5 out of 766), in which CAPSeq analysis of next-generation sequences identified heterozygous genotypes as homozygous, and specificity errors occurring 0.3% (2 out of 766) in which an alternate genotype was called (Table 2). Sensitivity errors also referred to as “allele dropout” result from unbalanced amplification of *HLA* alleles, exhibit low Call Rates, and most likely result from nucleotide polymorphisms that influence amplification efficiency [26], [27]. This can lead to reporting of an individual as homozygous due to the resulting unbalanced representation of the two alleles. Inspection of the next-generation sequencing data indicated that in the examples identified as persistent sensitivity errors (Table 2) the alleles were represented in the sequence data at unequal frequencies and incorporation of the Bagging approach resulted in improved sensitivity (Figure 2). As illustrated in the figure, the frequency of the “minor” sequence (occurring when *HLA* alleles are PCR amplified in an unbalanced manner) was detectable at improved sensitivity when Bagging iterations were increased. For example, increasing Bagging iterations improved sensitivity thresholds for the underrepresented allele from 15.6% for Bagging 5 to 8.8% when Bagging was performed 60 times. Moreover, the underlying data supported the genotype call obtained by the CAPSeq method in the sense that the non-called allele was the next most frequently identifiable sequence, presumably a reflection of unbalanced amplification rather than an effect of the CAPSeq application *per se*.

In contrast, persistent specificity errors were reported for 2 subjects (Table 2). In each instance inspection of individual sequences was consistent with the CAPSeq called genotype. For example, Subject 150 was identified as *HLA-DRB1* *03∶05, *11∶01 versus *HLA-DRB1* *03∶01:01G, *11∶01:01G while Subject 307 was genotyped as *HLA-DRB1* *11∶04, *13∶01 versus *HLA-DRB1* *11∶01:01G, *13∶01:01G by SSO and CAPSeq based methods, respectively (Supplementary Table S1). The CAPSeq identified genotype was strongly supported by the underlying sequence data accounting for 47% (236 out of 500) and 45% (157 out of 350) of the total number of sequences obtained from Subjects 150 and 307, and indicating the likely possibility that CAPSeq provided genotypes are, in fact, correct for these samples.

**Figure 3:**
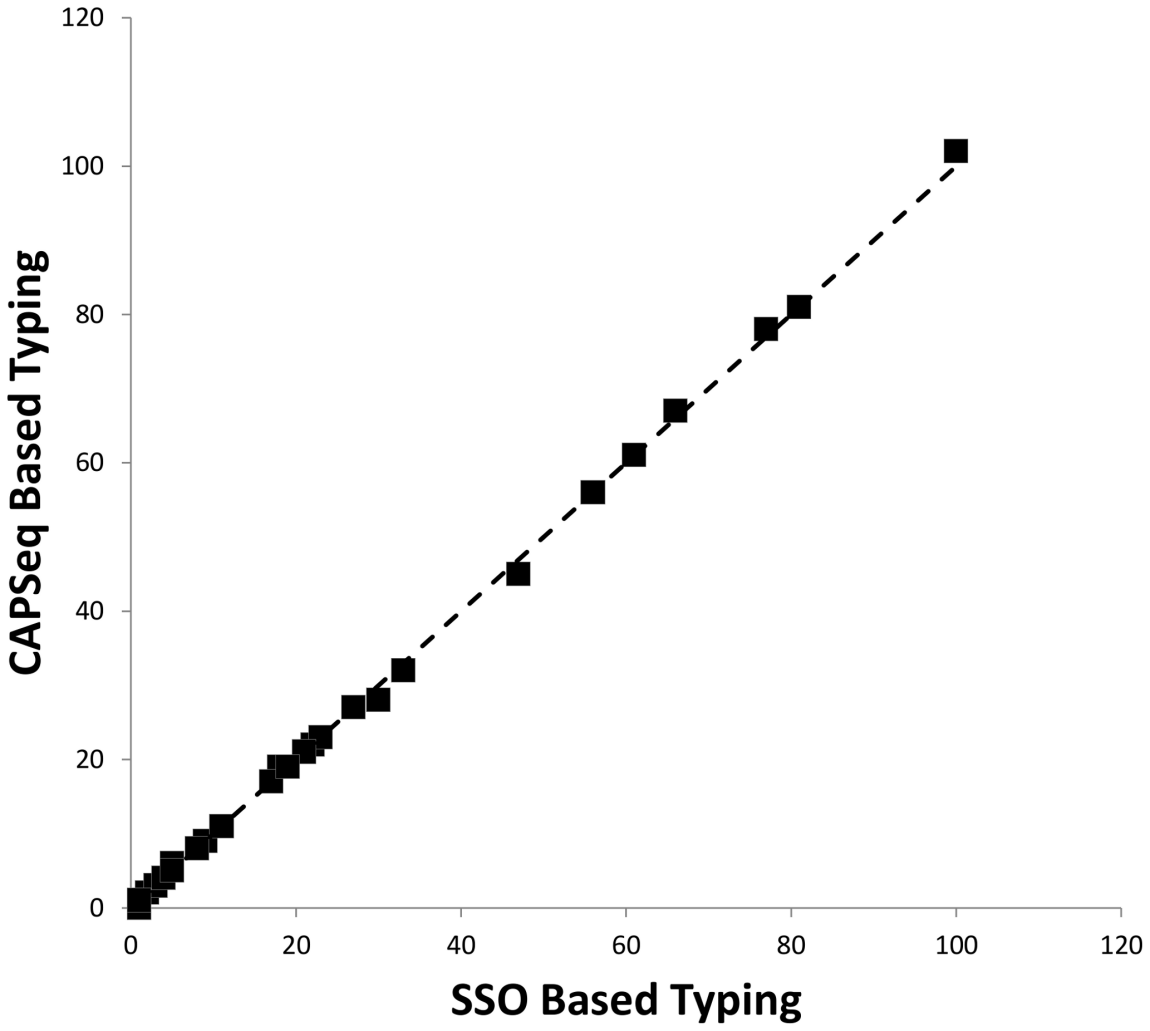
Frequency of *HLA-DRB1* genotypes obtained using SSO (x-axis) and CAPSeq (y-axis) compared at 4-digit resolution. The dashed line represents the theoretical identity between the two methods. Pearson’s correlation coefficient (r) exceeded 0.999.

The overall quality of the genotyping results were evaluated by comparing the frequency of alleles observed when analyzing next-generation sequencing data against the expected frequency determined by the SSO genotyping method (Figure 3). For example, the frequency of genotypes determined using the SSO and CAPSeq methods exhibited strong correlation with a Pearson’s correlation coefficient r>0.999 when the SSO genotypes and Bagging 60 results were compared (Figure 3). Moreover, as summarized in Table 1, the observed frequency of individual CAPSeq determined alleles were highly correlated with the expected frequencies of these *HLA-DRB1* alleles observed for subjects of European ancestry and to a lesser extent with worldwide allele frequencies, exhibiting Pearson’s correlation coefficients r>0.97 and 0.75, consistent with the expected validity of the CAPSeq analysis approach.

## Discussion

The *HLA* loci constitute the most highly polymorphic genetic system in humans [2]. A stable, inherited polymorphism, the alleles, gives rise to alternative forms of the protein with 5,764 variants reported [3]. The molecular basis for the *HLA* polymorphism resides in nucleotide sequence differences present in the coding regions of the HLA gene. Nearly all the *HLA* loci have various alleles with *HLA-DRB1* reported to have 1,260 distinct forms [3]. During recent years, substantial progress has been made in converting the protocols for molecular *HLA* genotyping to next-generation sequencing technologies [28]. For example, the choice of the Roche/454 GSFLX instrument allows direct sequencing of polymorphic *HLA* exons without the need to assemble partial reads. When compared with the SSO-based method, the next-generation sequencing approach to *HLA* genotyping provides increased accuracy and sample throughput [28]. Moreover, a number of publications have reported achieving high resolution typing of *HLA* loci using genomic DNA isolated from human cell lines, blood samples, as well as from cDNA created from isolations of mRNA [9], [10], [12], [14].

The CAPSeq software application was developed as an open source solution for genotyping of *HLA* alleles, offering a standalone approach to *HLA* genotyping. The CAPSeq analysis method has been developed initially for genotyping *-DRB1* alleles but has also been used with *-DPB1* providing similar levels of accuracy (data not shown). In contrast, computational methods for analysis of next-generation sequencing data generated on the Roche/454 GSFLX instrument are commercially available [17], [28]. In addition, use of the Genome Analysis Tool Kit (GATK) suite of open source software applications for *HLA* typing has also been described [16] and is available as source code but unfortunately is no longer supported by the GATK help desk [29]. Recently, Illumina based next-generation sequencing of *HLA* loci has also been reported with similar levels of accuracy [30]. Like CAPSeq, these various methods for analysis of next-generation sequences for *HLA* genotypes have reported a roughly 99% concordance when compared with test samples [16], [17], a threshold that may increase when subjects are examined directly via cloning and traditional Sanger based sequencing. The CAPSeq application uses the depth of sequencing provided by the next-generation based method to enable the R module diffusionMap in order to classify sequences based upon their similarity score. This feature results in the classification of sequences into their respective alleles. Along with MUMmer, used to align sequences, the application generates a consensus for each sequence class. The consensus sequence is used to identify known *HLA* genotypes with sequence identity scores approaching 100%. The inclusion of Bagging improves the frequency of concordant calls in excess of 99% when compared with the results of SSO based typing. The application is freely available (http://wpicr.wpic.pitt.edu/WPICCompGen/) and consists of R and PERL scripts as well as sample data and allele reference files for *HLA-DRB1*.

## Supporting Information

Table S1 ***HLA-DRB1*** **genotypes determined by SSO and CAPSeq analyses of next-generation sequencing data.** The table lists the Subject Identifier (column 1); Classification whether the SSO and CAPSeq results were concordant or discordant (column 2); Description of the error type or genotype (column 3); the 4-digit and 6-digit SSO and CAPSeq derived genotypes (columns 4 through 7); along with the CAPSeq derived values for the frequency with which individual alleles were called during the Bagging steps, Call Rate (columns 8 and 9), the average number of times each allele was observed when sampling the sequence data, Average Cluster Size (columns 10 and 11); and how closely each CAPSeq derived consensus sequence matched the sequence of the closest known *HLA-DRB1* allele, Average Identity Score (Columns 12 and 13).
(PDF)
