# Supplementary material for: Clustering and Alignment of Polymorphic Sequences for HLA-DRB1 Genotyping
Source: PLoS One. 2013 Mar 28;8(3):e59835. doi: 10.1371/journal.pone.0059835 (PMC3610899; doi:10.1371/journal.pone.0059835)
Supplement: Table S1 — HLA-DRB1 genotypes determined by SSO and CAPSeq analyses of next-generation sequencing data. The table lists the Subject Identifier (column 1); Classification whether the SSO and CAPSeq results were concordant or discordant (column 2); Description of the error type or genotype (column 3); the 4-digit and 6-digit SSO and CAPSeq derived genotypes (columns 4 through 7); along with the CAPSeq derived values for the frequency with which individual alleles were called during the Bagging steps, Call Rate (columns 8 and 9), the average number of times each allele was observed when sampling the sequence data, Average Cluster Size (columns 10 and 11); and how closely each CAPSeq derived consensus sequence matched the sequence of the closest known HLA-DRB1 allele, Average Identity Score (Columns 12 and 13). (PDF) [file pone.0059835.s001.pdf]

Supplementary Table 1. HLA-DRB1 genotypes

			SSO Genotype (4-Digit Resolution)		CAPSeq Genotype (6-Digit Resolution)		CAPSeq Call Rate		CAPSeq Avg. Cluster Size		CAPSeq Avg. Identity Score	
Subject_ID	Classification	Description	Allele 1	Allele 2	Allele 1	Allele 2	Allele 1	Allele 2	Allele 1	Allele 2	Allele 1	Allele 2
150	Discordant	Specificity	**DRB1*03:05**	DRB1*11:01	**DRB1*03:01:01G/03:01:11/03:50/03:68N**	DRB1*11:01:01G/11:01:02/11:01:06/11:100	105.0	98.3	45	43	100.0	100.0
307	Discordant	Specificity	**DRB1*11:04**	DRB1*13:01	**DRB1*11:01:01G/11:01:02/11:01:06/11:100**	DRB1*13:01:01G/13:01:08/13:105/13:112	100.0	83.3	60	36	100.0	100.0
228	Discordant	Sensitivity	DRB1*01:03	**DRB1*13:02**	DRB1*01:03	**DRB1*01:03**	100.0	100.0	89	89	100.0	100.0
277	Discordant	Sensitivity	**DRB1*11:04**	DRB1*16:01	**DRB1*16:01:01**	DRB1*16:01:01	100.0	100.0	89	89	100.0	100.0
306	Discordant	Sensitivity	DRB1*04:07	**DRB1*13:02**	DRB1*04:07:01G/04:07:03	**DRB1*04:07:01G/04:07:03**	100.0	100.0	91	91	100.0	100.0
337	Discordant	Sensitivity	**DRB1*13:01**	DRB1*15:01	**DRB1*15:01:01G**	DRB1*15:01:01G	100.0	100.0	87	87	100.0	100.0
360	Discordant	Sensitivity	**DRB1*13:01**	DRB1*15:01	**DRB1*15:01:01G**	DRB1*15:01:01G	100.0	100.0	83	83	100.0	100.0
1	Concordant	Heterozygous	DRB1*03:01	DRB1*13:01	DRB1*03:01:01G/03:01:11/03:50/03:68N	DRB1*13:01:01G/13:01:08/13:105/13:112	101.7	5.0	80	30	100.0	100.0
2	Concordant	Heterozygous	DRB1*01:03	DRB1*04:01	DRB1*01:03	DRB1*04:01:01/04:01:08	101.7	100.0	49	42	100.0	100.0
3	Concordant	Heterozygous	DRB1*01:01	DRB1*04:08	DRB1*01:01:01/01:01:05	DRB1*04:08:01	110.0	101.7	39	41	100.0	100.0
4	Concordant	Heterozygous	DRB1*03:01	DRB1*04:01	DRB1*03:01:01G/03:01:11/03:50/03:68N	DRB1*04:01:01/04:01:08	100.0	98.3	46	49	100.0	100.0
5	Concordant	Heterozygous	DRB1*07:01	DRB1*15:01	DRB1*07:01:01G	DRB1*15:01:01G	100.0	95.0	51	38	100.0	100.0
6	Concordant	Heterozygous	DRB1*01:01	DRB1*13:02	DRB1*01:01:01/01:01:05	DRB1*13:02:01/13:109/13:128	100.0	25.0	76	27	100.0	100.0
7	Concordant	Heterozygous	DRB1*04:04	DRB1*15:01	DRB1*04:04:01/04:23	DRB1*15:01:01G	91.7	100.0	35	57	100.0	100.0
8	Concordant	Heterozygous	DRB1*01:02	DRB1*15:01	DRB1*01:02:01	DRB1*15:01:01G	100.0	83.3	57	37	100.0	100.0
9	Concordant	Heterozygous	DRB1*07:01	DRB1*13:01	DRB1*07:01:01G	DRB1*13:01:01G/13:01:08/13:105/13:112	101.7	85.0	57	35	100.0	100.0
10	Concordant	Heterozygous	DRB1*04:07	DRB1*15:01	DRB1*04:07:01G/04:07:03	DRB1*15:01:01G	98.3	100.0	44	49	100.0	100.0
11	Concordant	Heterozygous	DRB1*01:03	DRB1*11:01	DRB1*01:03	DRB1*11:01:01G/11:01:02/11:01:06/11:100	101.7	81.7	59	33	100.0	100.0
12	Concordant	Heterozygous	DRB1*11:01	DRB1*13:01	DRB1*11:01:01G/11:01:02/11:01:06/11:100	DRB1*13:01:01G/13:01:08/13:105/13:112	100.0	93.3	54	35	100.0	100.0
13	Concordant	Heterozygous	DRB1*11:03	DRB1*15:01	DRB1*11:03	DRB1*15:01:01G	53.3	100.0	30	72	100.0	100.0
14	Concordant	Heterozygous	DRB1*03:01	DRB1*12:01	DRB1*03:01:01G/03:01:11/03:50/03:68N	DRB1*12:01:01G	101.7	100.0	38	50	100.0	100.0
15	Concordant	Heterozygous	DRB1*12:01	DRB1*15:02	DRB1*12:01:01G	DRB1*15:02:01/15:19	100.0	93.3	55	38	100.0	100.0
16	Concordant	Heterozygous	DRB1*09:01	DRB1*11:01	DRB1*09:01:02/09:09	DRB1*11:01:01G/11:01:02/11:01:06/11:100	98.3	93.3	57	38	100.0	100.0
17	Concordant	Heterozygous	DRB1*04:01	DRB1*13:02	DRB1*04:01:01/04:01:08	DRB1*13:02:01/13:109/13:128	101.7	73.3	63	32	100.0	100.0
18	Concordant	Heterozygous	DRB1*11:01	DRB1*13:01	DRB1*11:01:01G/11:01:02/11:01:06/11:100	DRB1*13:01:01G/13:01:08/13:105/13:112	101.7	93.3	55	36	100.0	100.0
19	Concordant	Heterozygous	DRB1*11:01	DRB1*15:01	DRB1*11:01:01G/11:01:02/11:01:06/11:100	DRB1*15:01:01G	51.7	100.0	29	66	99.9	100.0
20	Concordant	Heterozygous	DRB1*04:08	DRB1*07:01	DRB1*04:08:01	DRB1*07:01:01G	93.3	101.7	40	47	100.0	100.0
22	Concordant	Heterozygous	DRB1*08:01	DRB1*14:01	DRB1*08:01:01G/08:01:05/08:39	DRB1*14:01:01G/14:113/14:114	101.7	100.0	46	44	100.0	100.0
24	Concordant	Heterozygous	DRB1*12:01	DRB1*15:01	DRB1*12:01:01G	DRB1*15:01:01G	85.0	100.0	34	61	100.0	100.0
25	Concordant	Heterozygous	DRB1*07:01	DRB1*15:01	DRB1*07:01:01G	DRB1*15:01:01G	100.0	100.0	42	47	100.0	100.0
27	Concordant	Heterozygous	DRB1*04:01	DRB1*15:01	DRB1*04:01:01/04:01:08	DRB1*15:01:01G	100.0	96.7	48	44	100.0	100.0
28	Concordant	Heterozygous	DRB1*07:01	DRB1*13:01	DRB1*07:01:01G	DRB1*13:01:01G/13:01:08/13:105/13:112	106.7	78.3	55	34	100.0	100.0
29	Concordant	Heterozygous	DRB1*07:01	DRB1*13:01	DRB1*07:01:01G	DRB1*13:01:01G/13:01:08/13:105/13:112	103.3	96.7	48	39	100.0	100.0
30	Concordant	Heterozygous	DRB1*04:04	DRB1*15:01	DRB1*04:04:01/04:23	DRB1*15:01:01G	100.0	98.3	49	41	100.0	100.0
31	Concordant	Heterozygous	DRB1*11:01	DRB1*11:04	DRB1*11:01:01G/11:01:02/11:01:06/11:100	DRB1*11:04:01/11:04:02/11:04:06	53.7	46.3	93	92	99.9	100.0
32	Concordant	Heterozygous	DRB1*04:01	DRB1*13:02	DRB1*04:01:01/04:01:08	DRB1*13:02:01/13:109/13:128	100.0	53.3	70	33	100.0	100.0
33	Concordant	Heterozygous	DRB1*01:01	DRB1*16:01	DRB1*01:01:01/01:01:05	DRB1*16:01:01	103.3	100.0	40	47	100.0	100.0
34	Concordant	Heterozygous	DRB1*01:02	DRB1*13:03	DRB1*01:02:01	DRB1*13:03:01/13:03:02	105.0	90.0	54	37	100.0	100.0
35	Concordant	Heterozygous	DRB1*01:02	DRB1*15:01	DRB1*01:02:01	DRB1*15:01:01G	101.7	100.0	46	43	100.0	100.0
36	Concordant	Heterozygous	DRB1*03:01	DRB1*16:01	DRB1*03:01:01G/03:01:11/03:50/03:68N	DRB1*16:01:01	83.3	100.0	32	61	100.0	100.0
37	Concordant	Heterozygous	DRB1*11:04	DRB1*13:01	DRB1*11:04:01/11:04:02/11:04:06	DRB1*13:01:01G/13:01:08/13:105/13:112	100.0	96.7	58	35	100.0	100.0
38	Concordant	Heterozygous	DRB1*07:01	DRB1*15:01	DRB1*07:01:01G	DRB1*15:01:01G	95.0	100.0	38	54	100.0	100.0
39	Concordant	Heterozygous	DRB1*01:02	DRB1*08:01	DRB1*01:02:01	DRB1*08:01:01G/08:01:05/08:39	100.0	100.0	43	48	100.0	100.0
40	Concordant	Heterozygous	DRB1*03:01	DRB1*15:01	DRB1*03:01:01G/03:01:11/03:50/03:68N	DRB1*15:01:01G	80.0	101.7	33	60	100.0	100.0
41	Concordant	Heterozygous	DRB1*04:03	DRB1*13:01	DRB1*04:03:01/04:03:03/04:52	DRB1*13:01:01G/13:01:08/13:105/13:112	100.0	91.7	55	38	100.0	100.0
42	Concordant	Heterozygous	DRB1*11:01	DRB1*11:03	DRB1*11:01:01G/11:01:02/11:01:06/11:100	DRB1*11:03	60.0	60.0	66	72	100.0	100.0
43	Concordant	Heterozygous	DRB1*13:01	DRB1*16:01	DRB1*13:01:01G/13:01:08/13:105/13:112	DRB1*16:01:01	23.3	100.0	25	80	100.0	100.0
44	Concordant	Heterozygous	DRB1*11:01	DRB1*13:02	DRB1*11:01:01G/11:01:02/11:01:06/11:100	DRB1*13:02:01/13:109/13:128	100.0	83.3	61	33	100.0	100.0
45	Concordant	Heterozygous	DRB1*11:01	DRB1*15:01	DRB1*11:01:01G/11:01:02/11:01:06/11:100	DRB1*15:01:01G	100.0	100.0	44	49	100.0	100.0
47	Concordant	Heterozygous	DRB1*04:01	DRB1*08:01	DRB1*04:01:01/04:01:08	DRB1*08:01:01G/08:01:05/08:39	100.0	101.7	46	43	100.0	100.0
48	Concordant	Heterozygous	DRB1*11:01	DRB1*16:01	DRB1*11:01:01G/11:01:02/11:01:06/11:100	DRB1*16:01:01	43.3	100.0	30	74	100.0	100.0
49	Concordant	Heterozygous	DRB1*03:01	DRB1*07:01	DRB1*03:01:01G/03:01:11/03:50/03:68N	DRB1*07:01:01G	96.7	100.0	46	44	100.0	100.0
50	Concordant	Heterozygous	DRB1*01:01	DRB1*13:02	DRB1*01:01:01/01:01:05	DRB1*13:02:01/13:109/13:128	100.0	58.3	70	29	100.0	100.0
51	Concordant	Heterozygous	DRB1*10:01	DRB1*11:04	DRB1*10:01:01	DRB1*11:04:01/11:04:02/11:04:06	103.3	98.3	47	41	100.0	100.0
52	Concordant	Heterozygous	DRB1*04:04	DRB1*08:02	DRB1*04:04:01/04:23	DRB1*08:02:01/08:02:02	95.0	100.0	40	51	100.0	100.0
53	Concordant	Heterozygous	DRB1*01:01	DRB1*04:01	DRB1*01:01:01/01:01:05	DRB1*04:01:01/04:01:08	101.7	95.0	48	36	100.0	100.0
55	Concordant	Heterozygous	DRB1*01:01	DRB1*15:01	DRB1*01:01:01/01:01:05	DRB1*15:01:01G	100.0	90.0	53	41	100.0	100.0
56	Concordant	Heterozygous	DRB1*07:01	DRB1*11:04	DRB1*07:01:01G	DRB1*11:04:01/11:04:02/11:04:06	100.0	100.0	51	41	100.0	100.0
57	Concordant	Heterozygous	DRB1*04:04	DRB1*15:01	DRB1*04:04:01/04:23	DRB1*15:01:01G	98.3	100.0	43	51	100.0	100.0
58	Concordant	Heterozygous	DRB1*13:03	DRB1*15:01	DRB1*13:03:01/13:03:02	DRB1*15:01:01G	96.7	100.0	41	52	100.0	100.0
59	Concordant	Heterozygous	DRB1*07:01	DRB1*13:01	DRB1*07:01:01G	DRB1*13:01:01G/13:01:08/13:105/13:112	105.0	76.7	57	35	100.0	100.0
60	Concordant	Heterozygous	DRB1*01:01	DRB1*03:01	DRB1*01:01:01/01:01:05	DRB1*03:01:01G/03:01:11/03:50/03:68N	101.7	80.0	58	36	100.0	100.0
61	Concordant	Heterozygous	DRB1*04:04	DRB1*07:01	DRB1*04:04:01/04:23	DRB1*07:01:01G	98.3	98.3	48	40	100.0	100.0
62	Concordant	Heterozygous	DRB1*01:01	DRB1*11:01	DRB1*01:01:01/01:01:05	DRB1*11:01:01G/11:01:02/11:01:06/11:100	100.0	91.7	55	38	100.0	100.0
63	Concordant	Heterozygous	DRB1*11:01	DRB1*13:03	DRB1*11:01:01G/11:01:02/11:01:06/11:100	DRB1*13:03:01/13:03:02	98.3	98.3	47	48	100.0	100.0
64	Concordant	Heterozygous	DRB1*04:01	DRB1*15:01	DRB1*04:01:01/04:01:08	DRB1*15:01:01G	93.3	100.0	42	53	100.0	100.0
65	Concordant	Heterozygous	DRB1*07:01	DRB1*13:03	DRB1*07:01:01G	DRB1*13:03:01/13:03:02	101.7	100.0	42	47	100.0	100.0
66	Concordant	Heterozygous	DRB1*04:01	DRB1*14:01	DRB1*04:01:01/04:01:08	DRB1*14:01:01G/14:113/14:114	101.7	100.0	50	41	100.0	100.0
67	Concordant	Heterozygous	DRB1*10:01	DRB1*11:01	DRB1*10:01:01	DRB1*11:01:01G/11:01:02/11:01:06/11:100	98.3	96.7	53	39	100.0	100.0
68	Concordant	Heterozygous	DRB1*01:02	DRB1*15:01	DRB1*01:02:01	DRB1*15:01:01G	101.7	90.0	53	39	100.0	100.0

69	Concordant	Heterozygous	DRB1*01:02	DRB1*15:01	DRB1*01:02:01	DRB1*15:01:01G	103.3	85.0	56	36	100.0	100.0
70	Concordant	Heterozygous	DRB1*11:04	DRB1*15:01	DRB1*11:04:01/11:04:02/11:04:06	DRB1*15:01:01G	80.0	100.0	35	60	100.0	100.0
72	Concordant	Heterozygous	DRB1*04:01	DRB1*09:01	DRB1*04:01:01/04:01:08	DRB1*09:01:02/09:09	95.0	101.7	39	52	100.0	100.0
74	Concordant	Heterozygous	DRB1*03:01	DRB1*04:01	DRB1*03:01:01G/03:01:11/03:50/03:68N	DRB1*04:01:01/04:01:08	95.0	100.0	36	56	100.0	100.0
75	Concordant	Heterozygous	DRB1*04:04	DRB1*11:01	DRB1*04:04:01/04:23	DRB1*11:01:01G/11:01:02/11:01:06/11:100	100.0	30.0	76	28	100.0	100.0
76	Concordant	Heterozygous	DRB1*07:01	DRB1*15:01	DRB1*07:01:01G	DRB1*15:01:01G	93.3	100.0	37	54	100.0	100.0
77	Concordant	Heterozygous	DRB1*01:01	DRB1*07:01	DRB1*01:01:01/01:01:05	DRB1*07:01:01G	118.3	91.7	45	37	100.0	100.0
78	Concordant	Heterozygous	DRB1*04:01	DRB1*11:01	DRB1*04:01:01/04:01:08	DRB1*11:01:01G/11:01:02/11:01:06/11:100	100.0	32.2	75	31	100.0	100.0
79	Concordant	Heterozygous	DRB1*13:01	DRB1*15:01	DRB1*13:01:01G/13:01:08/13:105/13:112	DRB1*15:01:01G	18.3	100.0	27	81	100.0	100.0
80	Concordant	Heterozygous	DRB1*03:01	DRB1*08:10	DRB1*03:01:01G/03:01:11/03:50/03:68N	DRB1*08:10	100.0	101.7	40	47	100.0	100.0
81	Concordant	Heterozygous	DRB1*04:01	DRB1*07:01	DRB1*04:01:01/04:01:08	DRB1*07:01:01G	98.3	101.7	45	45	100.0	100.0
82	Concordant	Heterozygous	DRB1*04:01	DRB1*08:01	DRB1*04:01:01/04:01:08	DRB1*08:01:01G/08:01:05/08:39	100.0	90.0	55	40	100.0	100.0
83	Concordant	Heterozygous	DRB1*03:01	DRB1*07:01	DRB1*03:01:01G/03:01:11/03:50/03:68N	DRB1*07:01:01G	90.0	108.3	41	44	100.0	100.0
84	Concordant	Heterozygous	DRB1*07:01	DRB1*11:01	DRB1*07:01:01G	DRB1*11:01:01G/11:01:02/11:01:06/11:100	106.7	45.0	66	30	100.0	100.0
85	Concordant	Heterozygous	DRB1*03:01	DRB1*13:01	DRB1*03:01:01G/03:01:11/03:50/03:68N	DRB1*13:01:01G/13:01:08/13:105/13:112	100.0	15.0	81	26	100.0	100.0
86	Concordant	Heterozygous	DRB1*01:02	DRB1*03:01	DRB1*01:02:01	DRB1*03:01:01G/03:01:11/03:50/03:68N	113.3	90.0	44	40	100.0	100.0
87	Concordant	Heterozygous	DRB1*13:02	DRB1*15:01	DRB1*13:02:01/13:109/13:128	DRB1*15:01:01G	1.7	100.0	25	85	100.0	100.0
88	Concordant	Heterozygous	DRB1*12:01	DRB1*16:01	DRB1*12:01:01G	DRB1*16:01:01	95.0	100.0	47	39	100.0	100.0
89	Concordant	Heterozygous	DRB1*07:01	DRB1*15:01	DRB1*07:01:01G	DRB1*15:01:01G	100.0	98.3	45	45	100.0	100.0
90	Concordant	Heterozygous	DRB1*13:01	DRB1*15:01	DRB1*13:01:01G/13:01:08/13:105/13:112	DRB1*15:01:01G	21.7	100.0	27	81	100.0	100.0
91	Concordant	Heterozygous	DRB1*07:01	DRB1*08:01	DRB1*07:01:01G	DRB1*08:01:01G/08:01:05/08:39	93.3	111.7	41	44	100.0	100.0
92	Concordant	Heterozygous	DRB1*11:01	DRB1*13:02	DRB1*11:01:01G/11:01:02/11:01:06/11:100	DRB1*13:02:01/13:109/13:128	100.0	93.3	57	37	100.0	100.0
93	Concordant	Heterozygous	DRB1*10:01	DRB1*11:01	DRB1*10:01:01	DRB1*11:01:01G/11:01:02/11:01:06/11:100	101.7	100.0	42	47	100.0	100.0
94	Concordant	Heterozygous	DRB1*03:01	DRB1*04:01	DRB1*03:01:01G/03:01:11/03:50/03:68N	DRB1*04:01:01/04:01:08	98.3	100.0	45	47	100.0	100.0
95	Concordant	Heterozygous	DRB1*13:01	DRB1*13:02	DRB1*13:01:01G/13:01:08/13:105/13:112	DRB1*13:02:01/13:109/13:128	54.6	45.5	95	95	100.0	100.0
96	Concordant	Heterozygous	DRB1*03:01	DRB1*04:01	DRB1*03:01:01G/03:01:11/03:50/03:68N	DRB1*04:01:01/04:01:08	90.0	101.7	35	56	100.0	100.0
97	Concordant	Heterozygous	DRB1*04:05	DRB1*07:01	DRB1*04:05:01/04:05:03/04:05:04	DRB1*07:01:01G	105.0	96.7	47	41	100.0	100.0
98	Concordant	Heterozygous	DRB1*01:01	DRB1*10:01	DRB1*01:01:01/01:01:05	DRB1*10:01:01	103.3	95.0	49	41	100.0	100.0
99	Concordant	Heterozygous	DRB1*01:01	DRB1*09:01	DRB1*01:01:01/01:01:05	DRB1*09:01:02/09:09	105.0	93.3	46	41	100.0	100.0
100	Concordant	Heterozygous	DRB1*03:01	DRB1*07:01	DRB1*03:01:01G/03:01:11/03:50/03:68N	DRB1*07:01:01G	106.7	91.7	50	37	100.0	100.0
101	Concordant	Heterozygous	DRB1*01:02	DRB1*11:01	DRB1*01:02:01	DRB1*11:01:01G/11:01:02/11:01:06/11:100	100.0	73.3	61	34	100.0	100.0
103	Concordant	Heterozygous	DRB1*04:01	DRB1*15:01	DRB1*04:01:01/04:01:08	DRB1*15:01:01G	100.0	98.3	49	43	100.0	100.0
104	Concordant	Heterozygous	DRB1*11:01	DRB1*13:03	DRB1*11:01:01G/11:01:02/11:01:06/11:100	DRB1*13:03:01/13:03:02	86.7	100.0	36	59	100.0	100.0
105	Concordant	Heterozygous	DRB1*04:02	DRB1*13:02	DRB1*04:02:01	DRB1*13:02:01/13:109/13:128	103.3	33.3	69	29	100.0	100.0
106	Concordant	Heterozygous	DRB1*01:01	DRB1*11:01	DRB1*01:01:01/01:01:05	DRB1*11:01:01G/11:01:02/11:01:06/11:100	103.3	90.0	56	37	100.0	100.0
107	Concordant	Heterozygous	DRB1*01:01	DRB1*12:01	DRB1*01:01:01/01:01:05	DRB1*12:01:01G	101.7	100.0	41	48	100.0	100.0
108	Concordant	Heterozygous	DRB1*07:01	DRB1*13:02	DRB1*07:01:01G	DRB1*13:02:01/13:109/13:128	100.0	1.7	85	25	100.0	100.0
109	Concordant	Heterozygous	DRB1*11:04	DRB1*12:01	DRB1*11:04:01/11:04:02/11:04:06	DRB1*12:01:01G	58.3	105.0	32	64	100.0	100.0
110	Concordant	Heterozygous	DRB1*01:01	DRB1*11:01	DRB1*01:01:01/01:01:05	DRB1*11:01:01G/11:01:02/11:01:06/11:100	100.0	101.7	48	43	100.0	100.0
112	Concordant	Heterozygous	DRB1*01:01	DRB1*04:04	DRB1*01:01:01/01:01:05	DRB1*04:04:01/04:23	103.3	98.3	41	47	100.0	100.0
113	Concordant	Heterozygous	DRB1*04:01	DRB1*15:01	DRB1*04:01:01/04:01:08	DRB1*15:01:01G	98.3	100.0	45	48	100.0	100.0
114	Concordant	Heterozygous	DRB1*04:07	DRB1*09:01	DRB1*04:07:01G/04:07:03	DRB1*09:01:02/09:09	103.3	98.3	43	41	100.0	100.0
115	Concordant	Heterozygous	DRB1*03:01	DRB1*11:01	DRB1*03:01:01G/03:01:11/03:50/03:68N	DRB1*11:01:01G/11:01:02/11:01:06/11:100	108.3	88.3	51	39	100.0	100.0
116	Concordant	Heterozygous	DRB1*03:01	DRB1*07:01	DRB1*03:01:01G/03:01:11/03:50/03:68N	DRB1*07:01:01G	101.7	96.7	45	42	100.0	100.0
117	Concordant	Heterozygous	DRB1*04:03	DRB1*04:05	DRB1*04:03:01/04:03:03/04:52	DRB1*04:05:01/04:05:03/04:05:04	100.0	100.0	44	50	100.0	100.0
118	Concordant	Heterozygous	DRB1*12:01	DRB1*15:01	DRB1*12:01:01G	DRB1*15:01:01G	98.3	98.3	51	41	100.0	100.0
119	Concordant	Heterozygous	DRB1*03:01	DRB1*11:01	DRB1*03:01:01G/03:01:11/03:50/03:68N	DRB1*11:01:01G/11:01:02/11:01:06/11:100	103.3	88.3	54	39	100.0	100.0
121	Concordant	Heterozygous	DRB1*03:01	DRB1*15:01	DRB1*03:01:01G/03:01:11/03:50/03:68N	DRB1*15:01:01G	98.3	100.0	38	53	100.0	100.0
123	Concordant	Heterozygous	DRB1*01:03	DRB1*11:04	DRB1*01:03	DRB1*11:04:01/11:04:02/11:04:06	106.7	86.7	56	34	100.0	100.0
124	Concordant	Heterozygous	DRB1*09:01	DRB1*13:02	DRB1*09:01:02/09:09	DRB1*13:02:01/13:109/13:128	100.0	61.0	68	32	100.0	100.0
125	Concordant	Heterozygous	DRB1*07:01	DRB1*15:01	DRB1*07:01:01G	DRB1*15:01:01G	95.0	103.3	41	50	100.0	100.0
126	Concordant	Heterozygous	DRB1*01:01	DRB1*13:01	DRB1*01:01:01/01:01:05	DRB1*13:01:01G/13:01:08/13:105/13:112	100.0	42.4	73	29	100.0	100.0
127	Concordant	Heterozygous	DRB1*04:01	DRB1*11:01	DRB1*04:01:01/04:01:08	DRB1*11:01:01G/11:01:02/11:01:06/11:100	100.0	95.0	55	39	100.0	100.0
128	Concordant	Heterozygous	DRB1*01:02	DRB1*11:01	DRB1*01:02:01	DRB1*11:01:01G/11:01:02/11:01:06/11:100	101.7	80.0	59	35	100.0	100.0
129	Concordant	Heterozygous	DRB1*13:01	DRB1*14:01	DRB1*13:01:01G/13:01:08/13:105/13:112	DRB1*14:01:01G/14:113/14:114	88.3	100.0	40	55	100.0	100.0
130	Concordant	Heterozygous	DRB1*04:01	DRB1*08:01	DRB1*04:01:01/04:01:08	DRB1*08:01:01G/08:01:05/08:39	96.7	100.0	42	50	100.0	100.0
131	Concordant	Heterozygous	DRB1*11:01	DRB1*11:04	DRB1*11:01:01G/11:01:02/11:01:06/11:100	DRB1*11:04:01/11:04:02/11:04:06	72.3	27.7	93	94	100.0	100.0
132	Concordant	Heterozygous	DRB1*07:01	DRB1*16:01	DRB1*07:01:01G	DRB1*16:01:01	85.0	100.0	35	60	100.0	100.0
133	Concordant	Heterozygous	DRB1*11:01	DRB1*13:01	DRB1*11:01:01G/11:01:02/11:01:06/11:100	DRB1*13:01:01G/13:01:08/13:105/13:112	100.0	91.7	56	39	100.0	100.0
134	Concordant	Heterozygous	DRB1*13:01	DRB1*15:01	DRB1*13:01:01G/13:01:08/13:105/13:112	DRB1*15:01:01G	75.0	100.0	32	64	100.0	100.0
135	Concordant	Heterozygous	DRB1*14:01	DRB1*15:01	DRB1*14:01:01G/14:113/14:114	DRB1*15:01:01G	98.3	100.0	49	45	100.0	100.0
136	Concordant	Heterozygous	DRB1*04:01	DRB1*13:01	DRB1*04:01:01/04:01:08	DRB1*13:01:01G/13:01:08/13:105/13:112	100.0	83.3	63	33	100.0	100.0
137	Concordant	Heterozygous	DRB1*01:01	DRB1*12:01	DRB1*01:01:01/01:01:05	DRB1*12:01:01G	91.7	98.3	42	52	100.0	100.0
138	Concordant	Heterozygous	DRB1*01:03	DRB1*13:01	DRB1*01:03	DRB1*13:01:01G/13:01:08/13:105/13:112	100.0	16.1	83	28	100.0	100.0
139	Concordant	Heterozygous	DRB1*03:01	DRB1*13:01	DRB1*03:01:01G/03:01:11/03:50/03:68N	DRB1*13:01:01G/13:01:08/13:105/13:112	100.0	48.3	72	28	100.0	100.0
140	Concordant	Heterozygous	DRB1*03:01	DRB1*14:01	DRB1*03:01:01G/03:01:11/03:50/03:68N	DRB1*14:01:01G/14:113/14:114	101.7	100.0	43	49	100.0	100.0
143	Concordant	Heterozygous	DRB1*13:02	DRB1*15:01	DRB1*13:02:01/13:109/13:128	DRB1*15:01:01G	31.7	100.0	28	80	100.0	100.0
144	Concordant	Heterozygous	DRB1*07:01	DRB1*13:03	DRB1*07:01:01G	DRB1*13:03:01/13:03:02	101.7	20.0	80	28	100.0	100.0
145	Concordant	Heterozygous	DRB1*04:01	DRB1*15:01	DRB1*04:01:01/04:01:08	DRB1*15:01:01G	98.3	100.0	43	50	100.0	100.0
146	Concordant	Heterozygous	DRB1*07:01	DRB1*13:01	DRB1*07:01:01G	DRB1*13:01:01G/13:01:08/13:105/13:112	98.3	100.0	44	45	100.0	100.0
147	Concordant	Heterozygous	DRB1*08:01	DRB1*15:01	DRB1*08:01:01G/08:01:05/08:39	DRB1*15:01:01G	98.3	95.0	50	45	100.0	100.0
148	Concordant	Heterozygous	DRB1*04:01	DRB1*11:04	DRB1*04:01:01/04:01:08	DRB1*11:04:01/11:04:02/11:04:06	100.0	96.7	48	47	100.0	100.0
149	Concordant	Heterozygous	DRB1*13:01	DRB1*14:01	DRB1*13:01:01G/13:01:08/13:105/13:112	DRB1*14:01:01G/14:113/14:114	93.3	100.0	36	59	100.0	100.0

151	Concordant	Heterozygous	DRB1*01:01	DRB1*13:03	DRB1*01:01:01/01:01:05	DRB1*13:03:01/13:03:02	101.7	96.7	46	45	100.0	100.0
152	Concordant	Heterozygous	DRB1*15:01	DRB1*15:02	DRB1*15:01:01G	DRB1*15:02:01/15:19	50.0	50.0	89	89	100.0	99.8
153	Concordant	Heterozygous	DRB1*13:02	DRB1*16:01	DRB1*13:02:01/13:109/13:128	DRB1*16:01:01	88.3	100.0	34	60	100.0	100.0
154	Concordant	Heterozygous	DRB1*04:01	DRB1*11:01	DRB1*04:01:01/04:01:08	DRB1*11:01:01G/11:01:02/11:01:06/11:100	98.3	100.0	44	49	100.0	100.0
155	Concordant	Heterozygous	DRB1*07:01	DRB1*13:02	DRB1*07:01:01G	DRB1*13:02:01/13:109/13:128	106.7	90.0	52	37	100.0	100.0
156	Concordant	Heterozygous	DRB1*08:03	DRB1*12:01	DRB1*08:03:02/08:03:03	DRB1*12:01:01G	100.0	100.0	46	48	100.0	100.0
157	Concordant	Heterozygous	DRB1*01:03	DRB1*07:01	DRB1*01:03	DRB1*07:01:01G	103.3	88.3	56	37	100.0	100.0
158	Concordant	Heterozygous	DRB1*07:01	DRB1*12:01	DRB1*07:01:01G	DRB1*12:01:01G	71.7	100.0	32	62	100.0	100.0
159	Concordant	Heterozygous	DRB1*03:01	DRB1*11:04	DRB1*03:01:01G/03:01:11/03:50/03:68N	DRB1*11:04:01/11:04:02/11:04:06	106.7	98.3	41	46	100.0	100.0
160	Concordant	Heterozygous	DRB1*01:01	DRB1*13:01	DRB1*01:01:01/01:01:05	DRB1*13:01:01G/13:01:08/13:105/13:112	101.7	96.7	41	50	100.0	100.0
161	Concordant	Heterozygous	DRB1*04:04	DRB1*13:02	DRB1*04:04:01/04:23	DRB1*13:02:01/13:109/13:128	96.7	98.3	44	47	100.0	100.0
162	Concordant	Heterozygous	DRB1*11:01	DRB1*15:01	DRB1*11:01:01G/11:01:02/11:01:06/11:100	DRB1*15:01:01G	100.0	95.0	46	48	100.0	100.0
163	Concordant	Heterozygous	DRB1*01:03	DRB1*04:01	DRB1*01:03	DRB1*04:01:01/04:01:08	101.7	98.3	44	46	100.0	100.0
164	Concordant	Heterozygous	DRB1*01:01	DRB1*15:01	DRB1*01:01:01/01:01:05	DRB1*15:01:01G	98.3	100.0	43	47	100.0	100.0
165	Concordant	Heterozygous	DRB1*01:03	DRB1*13:02	DRB1*01:03	DRB1*13:02:01/13:109/13:128	106.7	100.0	40	47	100.0	100.0
166	Concordant	Heterozygous	DRB1*03:01	DRB1*13:01	DRB1*03:01:01G/03:01:11/03:50/03:68N	DRB1*13:01:01G/13:01:08/13:105/13:112	98.3	100.0	42	46	100.0	100.0
167	Concordant	Heterozygous	DRB1*03:01	DRB1*11:01	DRB1*03:01:01G/03:01:11/03:50/03:68N	DRB1*11:01:01G/11:01:02/11:01:06/11:100	103.3	100.0	40	49	100.0	100.0
168	Concordant	Heterozygous	DRB1*04:01	DRB1*07:01	DRB1*04:01:01/04:01:08	DRB1*07:01:01G	88.3	110.0	38	49	100.0	100.0
169	Concordant	Heterozygous	DRB1*03:01	DRB1*04:01	DRB1*03:01:01G/03:01:11/03:50/03:68N	DRB1*04:01:01/04:01:08	100.0	100.0	48	45	100.0	100.0
170	Concordant	Heterozygous	DRB1*04:07	DRB1*15:01	DRB1*04:07:01G/04:07:03	DRB1*15:01:01G	98.3	100.0	47	48	100.0	100.0
171	Concordant	Heterozygous	DRB1*03:01	DRB1*14:01	DRB1*03:01:01G/03:01:11/03:50/03:68N	DRB1*14:01:01G/14:113/14:114	98.3	100.0	43	48	100.0	100.0
172	Concordant	Heterozygous	DRB1*01:01	DRB1*04:01	DRB1*01:01:01/01:01:05	DRB1*04:01:01/04:01:08	106.7	98.3	44	38	100.0	100.0
173	Concordant	Heterozygous	DRB1*01:02	DRB1*11:01	DRB1*01:02:01	DRB1*11:01:01G/11:01:02/11:01:06/11:100	103.3	96.7	47	46	100.0	100.0
174	Concordant	Heterozygous	DRB1*08:01	DRB1*11:01	DRB1*08:01:01G/08:01:05/08:39	DRB1*11:01:01G/11:01:02/11:01:06/11:100	100.0	91.7	54	41	100.0	100.0
176	Concordant	Heterozygous	DRB1*07:01	DRB1*08:01	DRB1*07:01:01G	DRB1*08:01:01G/08:01:05/08:39	85.0	105.0	35	53	100.0	100.0
177	Concordant	Heterozygous	DRB1*04:03	DRB1*07:01	DRB1*04:03:01/04:03:03/04:52	DRB1*07:01:01G	98.3	100.0	38	50	100.0	100.0
178	Concordant	Heterozygous	DRB1*14:01	DRB1*15:01	DRB1*14:01:01G/14:113/14:114	DRB1*15:01:01G	100.0	96.7	47	47	100.0	100.0
179	Concordant	Heterozygous	DRB1*07:01	DRB1*11:01	DRB1*07:01:01G	DRB1*11:01:01G/11:01:02/11:01:06/11:100	98.3	105.0	44	45	100.0	100.0
180	Concordant	Heterozygous	DRB1*01:01	DRB1*15:01	DRB1*01:01:01/01:01:05	DRB1*15:01:01G	100.0	98.3	50	40	100.0	100.0
182	Concordant	Heterozygous	DRB1*08:01	DRB1*13:01	DRB1*08:01:01G/08:01:05/08:39	DRB1*13:01:01G/13:01:08/13:105/13:112	100.0	93.3	54	41	100.0	100.0
183	Concordant	Heterozygous	DRB1*04:01	DRB1*08:01	DRB1*04:01:01/04:01:08	DRB1*08:01:01G/08:01:05/08:39	95.0	100.0	38	54	100.0	100.0
184	Concordant	Heterozygous	DRB1*04:01	DRB1*14:01	DRB1*04:01:01/04:01:08	DRB1*14:01:01G/14:113/14:114	96.7	100.0	41	50	100.0	100.0
186	Concordant	Heterozygous	DRB1*07:01	DRB1*12:01	DRB1*07:01:01G	DRB1*12:01:01G	98.3	101.7	42	47	100.0	100.0
187	Concordant	Heterozygous	DRB1*07:01	DRB1*13:01	DRB1*07:01:01G	DRB1*13:01:01G/13:01:08/13:105/13:112	100.0	93.3	46	42	100.0	100.0
188	Concordant	Heterozygous	DRB1*04:01	DRB1*11:01	DRB1*04:01:01/04:01:08	DRB1*11:01:01G/11:01:02/11:01:06/11:100	100.0	100.0	43	49	100.0	100.0
189	Concordant	Heterozygous	DRB1*04:02	DRB1*09:01	DRB1*04:02:01	DRB1*09:01:02/09:09	90.0	101.7	35	52	100.0	100.0
190	Concordant	Heterozygous	DRB1*10:01	DRB1*15:01	DRB1*10:01:01	DRB1*15:01:01G	96.7	100.0	42	49	100.0	100.0
191	Concordant	Heterozygous	DRB1*11:01	DRB1*15:01	DRB1*11:01:01G/11:01:02/11:01:06/11:100	DRB1*15:01:01G	100.0	100.0	44	46	100.0	100.0
192	Concordant	Heterozygous	DRB1*03:01	DRB1*07:01	DRB1*03:01:01G/03:01:11/03:50/03:68N	DRB1*07:01:01G	90.0	100.0	35	53	100.0	100.0
193	Concordant	Heterozygous	DRB1*11:01	DRB1*11:29	DRB1*11:01:01G/11:01:02/11:01:06/11:100	DRB1*11:29	81.7	75.0	62	58	100.0	100.0
194	Concordant	Heterozygous	DRB1*04:01	DRB1*15:01	DRB1*04:01:01/04:01:08	DRB1*15:01:01G	98.3	100.0	42	52	100.0	100.0
195	Concordant	Heterozygous	DRB1*11:01	DRB1*12:01	DRB1*11:01:01G/11:01:02/11:01:06/11:100	DRB1*12:01:01G	101.7	98.3	44	47	100.0	100.0
196	Concordant	Heterozygous	DRB1*11:01	DRB1*14:01	DRB1*11:01:01G/11:01:02/11:01:06/11:100	DRB1*14:01:01G/14:113/14:114	98.3	98.3	46	46	100.0	100.0
197	Concordant	Heterozygous	DRB1*07:01	DRB1*15:01	DRB1*07:01:01G	DRB1*15:01:01G	101.7	96.7	51	37	100.0	100.0
198	Concordant	Heterozygous	DRB1*07:01	DRB1*13:02	DRB1*07:01:01G	DRB1*13:02:01/13:109/13:128	105.0	66.7	59	34	100.0	100.0
199	Concordant	Heterozygous	DRB1*04:01	DRB1*07:01	DRB1*04:01:01/04:01:08	DRB1*07:01:01G	85.0	105.0	34	55	100.0	100.0
200	Concordant	Heterozygous	DRB1*04:04	DRB1*07:01	DRB1*04:04:01/04:23	DRB1*07:01:01G	91.7	101.7	37	51	100.0	100.0
201	Concordant	Heterozygous	DRB1*08:01	DRB1*16:01	DRB1*08:01:01G/08:01:05/08:39	DRB1*16:01:01	100.0	91.7	54	39	100.0	100.0
202	Concordant	Heterozygous	DRB1*12:01	DRB1*15:01	DRB1*12:01:01G	DRB1*15:01:01G	101.7	98.3	51	42	100.0	100.0
203	Concordant	Heterozygous	DRB1*08:04	DRB1*11:04	DRB1*08:04:01/08:04:04	DRB1*11:04:01/11:04:02/11:04:06	98.3	96.7	47	45	100.0	100.0
204	Concordant	Heterozygous	DRB1*03:01	DRB1*04:05	DRB1*03:01:01G/03:01:11/03:50/03:68N	DRB1*04:05:01/04:05:03/04:05:04	93.3	96.7	47	46	100.0	100.0
206	Concordant	Heterozygous	DRB1*07:01	DRB1*10:01	DRB1*07:01:01G	DRB1*10:01:01	100.0	100.0	45	44	100.0	100.0
207	Concordant	Heterozygous	DRB1*07:01	DRB1*08:01	DRB1*07:01:01G	DRB1*08:01:01G/08:01:05/08:39	91.7	101.7	39	52	100.0	100.0
208	Concordant	Heterozygous	DRB1*03:01	DRB1*08:01	DRB1*03:01:01G/03:01:11/03:50/03:68N	DRB1*08:01:01G/08:01:05/08:39	88.3	103.3	42	49	100.0	100.0
209	Concordant	Heterozygous	DRB1*01:01	DRB1*13:01	DRB1*01:01:01/01:01:05	DRB1*13:01:01G/13:01:08/13:105/13:112	103.3	83.3	55	38	100.0	100.0
210	Concordant	Heterozygous	DRB1*07:01	DRB1*11:14	DRB1*07:01:01G	DRB1*11:14:01/11:14:02	100.0	96.7	48	42	100.0	100.0
211	Concordant	Heterozygous	DRB1*03:01	DRB1*11:01	DRB1*03:01:01G/03:01:11/03:50/03:68N	DRB1*11:01:01G/11:01:02/11:01:06/11:100	100.0	100.0	39	49	100.0	100.0
212	Concordant	Heterozygous	DRB1*03:01	DRB1*04:01	DRB1*03:01:01G/03:01:11/03:50/03:68N	DRB1*04:01:01/04:01:08	101.7	98.3	45	44	100.0	100.0
213	Concordant	Heterozygous	DRB1*07:01	DRB1*16:01	DRB1*07:01:01G	DRB1*16:01:01	100.0	98.3	48	44	100.0	100.0
214	Concordant	Heterozygous	DRB1*03:01	DRB1*04:01	DRB1*03:01:01G/03:01:11/03:50/03:68N	DRB1*04:01:01/04:01:08	95.0	108.3	39	49	100.0	100.0
215	Concordant	Heterozygous	DRB1*01:02	DRB1*11:15	DRB1*01:02:01	DRB1*11:15	103.3	98.3	51	38	100.0	100.0
216	Concordant	Heterozygous	DRB1*08:01	DRB1*11:01	DRB1*08:01:01G/08:01:05/08:39	DRB1*11:01:01G/11:01:02/11:01:06/11:100	100.0	83.3	61	35	100.0	100.0
217	Concordant	Heterozygous	DRB1*03:01	DRB1*13:27	DRB1*03:01:01G/03:01:11/03:50/03:68N	DRB1*13:27	100.0	16.7	77	30	100.0	100.0
218	Concordant	Heterozygous	DRB1*01:01	DRB1*04:04	DRB1*01:01:01/01:01:05	DRB1*04:04:01/04:23	126.7	96.7	32	45	100.0	100.0
219	Concordant	Heterozygous	DRB1*03:01	DRB1*11:01	DRB1*03:01:01G/03:01:11/03:50/03:68N	DRB1*11:01:01G/11:01:02/11:01:06/11:100	110.0	91.7	50	38	100.0	100.0
220	Concordant	Heterozygous	DRB1*07:01	DRB1*15:01	DRB1*07:01:01G	DRB1*15:01:01G	98.3	100.0	41	49	100.0	100.0
221	Concordant	Heterozygous	DRB1*07:01	DRB1*11:04	DRB1*07:01:01G	DRB1*11:04:01/11:04:02/11:04:06	100.0	98.3	48	43	100.0	100.0
222	Concordant	Heterozygous	DRB1*07:01	DRB1*13:02	DRB1*07:01:01G	DRB1*13:02:01/13:109/13:128	100.0	60.0	68	30	100.0	100.0
223	Concordant	Heterozygous	DRB1*04:01	DRB1*08:03	DRB1*04:01:01/04:01:08	DRB1*08:03:02/08:03:03	100.0	100.0	48	45	100.0	100.0
224	Concordant	Heterozygous	DRB1*11:01	DRB1*14:01	DRB1*11:01:01G/11:01:02/11:01:06/11:100	DRB1*14:01:01G/14:113/14:114	91.7	98.3	40	54	100.0	100.0
225	Concordant	Heterozygous	DRB1*07:01	DRB1*13:01	DRB1*07:01:01G	DRB1*13:01:01G/13:01:08/13:105/13:112	100.0	81.7	58	37	100.0	100.0
226	Concordant	Heterozygous	DRB1*01:01	DRB1*08:01	DRB1*01:01:01/01:01:05	DRB1*08:01:01G/08:01:05/08:39	88.3	101.7	43	50	100.0	100.0
227	Concordant	Heterozygous	DRB1*01:03	DRB1*12:01	DRB1*01:03	DRB1*12:01:01G	103.3	100.0	42	48	100.0	100.0

229	Concordant	Heterozygous	DRB1*07:01	DRB1*13:01	DRB1*07:01:01G	DRB1*13:01:01G/13:01:08/13:105/13:112	100.0	18.3	79	30	100.0	100.0
231	Concordant	Heterozygous	DRB1*09:01	DRB1*11:04	DRB1*09:01:02/09:09	DRB1*11:04:01/11:04:02/11:04:06	101.7	83.3	59	35	100.0	100.0
232	Concordant	Heterozygous	DRB1*01:01	DRB1*11:04	DRB1*01:01:01/01:01:05	DRB1*11:04:01/11:04:02/11:04:06	100.0	91.7	49	44	100.0	100.0
233	Concordant	Heterozygous	DRB1*07:01	DRB1*13:02	DRB1*07:01:01G	DRB1*13:02:01/13:109/13:128	105.0	90.0	52	39	100.0	100.0
234	Concordant	Heterozygous	DRB1*08:01	DRB1*09:01	DRB1*08:01:01G/08:01:05/08:39	DRB1*09:01:02/09:09	101.7	96.7	43	48	100.0	100.0
235	Concordant	Heterozygous	DRB1*10:01	DRB1*15:01	DRB1*10:01:01	DRB1*15:01:01G	98.3	96.7	47	44	100.0	100.0
236	Concordant	Heterozygous	DRB1*03:01	DRB1*08:01	DRB1*03:01:01G/03:01:11/03:50/03:68N	DRB1*08:01:01G/08:01:05/08:39	98.3	100.0	46	45	100.0	100.0
237	Concordant	Heterozygous	DRB1*12:01	DRB1*13:01	DRB1*12:01:01G	DRB1*13:01:01G/13:01:08/13:105/13:112	100.0	56.7	71	29	100.0	100.0
238	Concordant	Heterozygous	DRB1*04:01	DRB1*11:04	DRB1*04:01:01/04:01:08	DRB1*11:04:01/11:04:02/11:04:06	100.0	98.3	53	41	100.0	100.0
239	Concordant	Heterozygous	DRB1*08:04	DRB1*13:01	DRB1*08:04:01/08:04:04	DRB1*13:01:01G/13:01:08/13:105/13:112	100.0	90.0	58	35	100.0	100.0
240	Concordant	Heterozygous	DRB1*04:01	DRB1*13:02	DRB1*04:01:01/04:01:08	DRB1*13:02:01/13:109/13:128	100.0	88.3	59	37	100.0	100.0
241	Concordant	Heterozygous	DRB1*01:01	DRB1*15:01	DRB1*01:01:01/01:01:05	DRB1*15:01:01G	101.7	100.0	42	46	100.0	100.0
242	Concordant	Heterozygous	DRB1*15:01	DRB1*16:01	DRB1*15:01:01G	DRB1*16:01:01	98.3	100.0	43	47	100.0	100.0
243	Concordant	Heterozygous	DRB1*07:01	DRB1*11:01	DRB1*07:01:01G	DRB1*11:01:01G/11:01:02/11:01:06/11:100	100.0	93.3	54	38	100.0	100.0
244	Concordant	Heterozygous	DRB1*01:02	DRB1*01:03	DRB1*01:02:01	DRB1*01:03	100.0	98.3	48	44	100.0	100.0
245	Concordant	Heterozygous	DRB1*01:01	DRB1*01:02	DRB1*01:01:01/01:01:05	DRB1*01:02:01	98.3	100.0	44	51	100.0	100.0
246	Concordant	Heterozygous	DRB1*01:01	DRB1*07:01	DRB1*01:01:01/01:01:05	DRB1*07:01:01G	105.0	86.7	51	36	100.0	100.0
248	Concordant	Heterozygous	DRB1*01:02	DRB1*11:04	DRB1*01:02:01	DRB1*11:04:01/11:04:02/11:04:06	101.7	91.7	55	37	100.0	100.0
249	Concordant	Heterozygous	DRB1*01:01	DRB1*15:01	DRB1*01:01:01/01:01:05	DRB1*15:01:01G	98.3	95.0	50	41	100.0	100.0
250	Concordant	Heterozygous	DRB1*08:01	DRB1*13:01	DRB1*08:01:01G/08:01:05/08:39	DRB1*13:01:01G/13:01:08/13:105/13:112	100.0	68.3	65	33	100.0	100.0
251	Concordant	Heterozygous	DRB1*07:01	DRB1*15:01	DRB1*07:01:01G	DRB1*15:01:01G	98.3	98.3	51	39	100.0	100.0
252	Concordant	Heterozygous	DRB1*01:01	DRB1*04:01	DRB1*01:01:01/01:01:05	DRB1*04:01:01/04:01:08	103.3	98.3	43	41	100.0	100.0
253	Concordant	Heterozygous	DRB1*01:03	DRB1*15:01	DRB1*01:03	DRB1*15:01:01G	103.3	96.7	51	38	100.0	100.0
254	Concordant	Heterozygous	DRB1*03:01	DRB1*15:01	DRB1*03:01:01G/03:01:11/03:50/03:68N	DRB1*15:01:01G	100.0	100.0	41	51	100.0	100.0
257	Concordant	Heterozygous	DRB1*03:01	DRB1*13:01	DRB1*03:01:01G/03:01:11/03:50/03:68N	DRB1*13:01:01G/13:01:08/13:105/13:112	103.3	85.0	53	34	100.0	100.0
258	Concordant	Heterozygous	DRB1*11:01	DRB1*11:04	DRB1*11:01:01G/11:01:02/11:01:06/11:100	DRB1*11:04:01/11:04:02/11:04:06	38.6	61.4	92	93	99.9	100.0
259	Concordant	Heterozygous	DRB1*04:01	DRB1*14:01	DRB1*04:01:01/04:01:08	DRB1*14:01:01G/14:113/14:114	93.3	105.0	41	49	100.0	100.0
260	Concordant	Heterozygous	DRB1*01:01	DRB1*16:01	DRB1*01:01:01/01:01:05	DRB1*16:01:01	100.0	98.3	41	50	100.0	100.0
261	Concordant	Heterozygous	DRB1*08:04	DRB1*13:01	DRB1*08:04:01/08:04:04	DRB1*13:01:01G/13:01:08/13:105/13:112	101.7	91.7	55	37	100.0	100.0
262	Concordant	Heterozygous	DRB1*04:05	DRB1*15:01	DRB1*04:05:01/04:05:03/04:05:04	DRB1*15:01:01G	100.0	98.3	51	42	100.0	100.0
263	Concordant	Heterozygous	DRB1*03:01	DRB1*04:01	DRB1*03:01:01G/03:01:11/03:50/03:68N	DRB1*04:01:01/04:01:08	98.3	100.0	49	44	100.0	100.0
264	Concordant	Heterozygous	DRB1*01:01	DRB1*07:01	DRB1*01:01:01/01:01:05	DRB1*07:01:01G	105.0	81.7	53	37	100.0	100.0
265	Concordant	Heterozygous	DRB1*07:01	DRB1*11:01	DRB1*07:01:01G	DRB1*11:01:01G/11:01:02/11:01:06/11:100	111.7	81.7	53	33	100.0	100.0
267	Concordant	Heterozygous	DRB1*13:01	DRB1*16:01	DRB1*13:01:01G/13:01:08/13:105/13:112	DRB1*16:01:01	66.7	101.7	30	66	100.0	100.0
268	Concordant	Heterozygous	DRB1*03:01	DRB1*07:01	DRB1*03:01:01G/03:01:11/03:50/03:68N	DRB1*07:01:01G	80.0	111.7	37	45	100.0	100.0
269	Concordant	Heterozygous	DRB1*07:01	DRB1*15:01	DRB1*07:01:01G	DRB1*15:01:01G	95.0	100.0	43	49	100.0	100.0
270	Concordant	Heterozygous	DRB1*03:01	DRB1*11:04	DRB1*03:01:01G/03:01:11/03:50/03:68N	DRB1*11:04:01/11:04:02/11:04:06	106.7	95.0	51	37	100.0	100.0
272	Concordant	Heterozygous	DRB1*11:04	DRB1*12:01	DRB1*11:04:01/11:04:02/11:04:06	DRB1*12:01:01G	73.3	100.0	31	66	100.0	100.0
273	Concordant	Heterozygous	DRB1*15:01	DRB1*16:01	DRB1*15:01:01G	DRB1*16:01:01	93.3	100.0	39	52	100.0	100.0
274	Concordant	Heterozygous	DRB1*01:01	DRB1*13:01	DRB1*01:01:01/01:01:05	DRB1*13:01:01G/13:01:08/13:105/13:112	100.0	93.3	54	39	100.0	100.0
275	Concordant	Heterozygous	DRB1*01:01	DRB1*04:01	DRB1*01:01:01/01:01:05	DRB1*04:01:01/04:01:08	103.3	93.3	48	40	100.0	100.0
276	Concordant	Heterozygous	DRB1*03:01	DRB1*13:02	DRB1*03:01:01G/03:01:11/03:50/03:68N	DRB1*13:02:01/13:109/13:128	100.0	68.3	63	32	100.0	100.0
278	Concordant	Heterozygous	DRB1*04:01	DRB1*15:01	DRB1*04:01:01/04:01:08	DRB1*15:01:01G	98.3	105.0	42	47	100.0	100.0
279	Concordant	Heterozygous	DRB1*11:01	DRB1*11:02	DRB1*11:01:01G/11:01:02/11:01:06/11:100	DRB1*11:02:01	80.0	68.3	60	53	100.0	100.0
280	Concordant	Heterozygous	DRB1*15:01	DRB1*15:02	DRB1*15:01:01G	DRB1*15:02:01/15:19	58.9	41.1	90	89	100.0	100.0
281	Concordant	Heterozygous	DRB1*03:01	DRB1*11:01	DRB1*03:01:01G/03:01:11/03:50/03:68N	DRB1*11:01:01G/11:01:02/11:01:06/11:100	101.7	93.3	48	44	100.0	100.0
282	Concordant	Heterozygous	DRB1*01:01	DRB1*07:01	DRB1*01:01:01/01:01:05	DRB1*07:01:01G	103.3	83.3	55	36	100.0	100.0
284	Concordant	Heterozygous	DRB1*01:01	DRB1*15:01	DRB1*01:01:01/01:01:05	DRB1*15:01:01G	100.0	95.0	51	39	100.0	100.0
285	Concordant	Heterozygous	DRB1*01:01	DRB1*01:03	DRB1*01:01:01/01:01:05	DRB1*01:03	100.0	100.0	44	48	100.0	100.0
286	Concordant	Heterozygous	DRB1*01:01	DRB1*04:04	DRB1*01:01:01/01:01:05	DRB1*04:04:01/04:23	105.0	90.0	51	36	100.0	100.0
288	Concordant	Heterozygous	DRB1*01:02	DRB1*15:02	DRB1*01:02:01	DRB1*15:02:01/15:19	100.0	100.0	48	43	100.0	100.0
289	Concordant	Heterozygous	DRB1*03:01	DRB1*12:01	DRB1*03:01:01G/03:01:11/03:50/03:68N	DRB1*12:01:01G	111.7	100.0	41	43	100.0	100.0
290	Concordant	Heterozygous	DRB1*11:01	DRB1*16:01	DRB1*11:01:01G/11:01:02/11:01:06/11:100	DRB1*16:01:01	10.0	100.0	27	84	100.0	100.0
291	Concordant	Heterozygous	DRB1*14:01	DRB1*16:01	DRB1*14:01:01G/14:113/14:114	DRB1*16:01:01	83.3	100.0	34	60	100.0	100.0
292	Concordant	Heterozygous	DRB1*11:01	DRB1*15:01	DRB1*11:01:01G/11:01:02/11:01:06/11:100	DRB1*15:01:01G	61.7	100.0	31	70	100.0	100.0
293	Concordant	Heterozygous	DRB1*08:01	DRB1*13:03	DRB1*08:01:01G/08:01:05/08:39	DRB1*13:03:01/13:03:02	100.0	58.3	63	31	100.0	100.0
294	Concordant	Heterozygous	DRB1*13:01	DRB1*13:03	DRB1*13:01:01G/13:01:08/13:105/13:112	DRB1*13:03:01/13:03:02	95.0	100.0	36	56	100.0	100.0
295	Concordant	Heterozygous	DRB1*03:01	DRB1*15:01	DRB1*03:01:01G/03:01:11/03:50/03:68N	DRB1*15:01:01G	101.7	100.0	42	50	100.0	100.0
296	Concordant	Heterozygous	DRB1*01:01	DRB1*11:04	DRB1*01:01:01/01:01:05	DRB1*11:04:01/11:04:02/11:04:06	105.0	70.0	61	32	100.0	100.0
297	Concordant	Heterozygous	DRB1*01:03	DRB1*04:01	DRB1*01:03	DRB1*04:01:01/04:01:08	101.7	88.3	55	36	100.0	100.0
299	Concordant	Heterozygous	DRB1*01:01	DRB1*08:01	DRB1*01:01:01/01:01:05	DRB1*08:01:01G/08:01:05/08:39	103.3	71.7	61	33	100.0	100.0
301	Concordant	Heterozygous	DRB1*01:02	DRB1*11:01	DRB1*01:02:01	DRB1*11:01:01G/11:01:02/11:01:06/11:100	100.0	21.7	82	27	100.0	100.0
303	Concordant	Heterozygous	DRB1*03:01	DRB1*04:01	DRB1*03:01:01G/03:01:11/03:50/03:68N	DRB1*04:01:01/04:01:08	96.7	98.3	44	48	100.0	100.0
304	Concordant	Heterozygous	DRB1*13:01	DRB1*14:01	DRB1*13:01:01G/13:01:08/13:105/13:112	DRB1*14:01:01G/14:113/14:114	61.7	100.0	28	70	100.0	100.0
308	Concordant	Heterozygous	DRB1*13:02	DRB1*15:01	DRB1*13:02:01/13:109/13:128	DRB1*15:01:01G	46.7	100.0	28	73	100.0	100.0
309	Concordant	Heterozygous	DRB1*01:01	DRB1*11:01	DRB1*01:01:01/01:01:05	DRB1*11:01:01G/11:01:02/11:01:06/11:100	103.3	46.7	69	29	100.0	100.0
310	Concordant	Heterozygous	DRB1*03:01	DRB1*04:01	DRB1*03:01:01G/03:01:11/03:50/03:68N	DRB1*04:01:01/04:01:08	96.7	100.0	47	46	100.0	100.0
311	Concordant	Heterozygous	DRB1*03:01	DRB1*15:01	DRB1*03:01:01G/03:01:11/03:50/03:68N	DRB1*15:01:01G	103.3	95.0	48	42	100.0	100.0
312	Concordant	Heterozygous	DRB1*01:02	DRB1*07:01	DRB1*01:02:01	DRB1*07:01:01G	105.4	28.6	74	28	100.0	100.0
313	Concordant	Heterozygous	DRB1*04:04	DRB1*13:02	DRB1*04:04:01/04:23	DRB1*13:02:01/13:109/13:128	100.0	35.0	74	30	100.0	100.0
314	Concordant	Heterozygous	DRB1*10:01	DRB1*15:01	DRB1*10:01:01	DRB1*15:01:01G	95.0	101.7	40	51	100.0	100.0
315	Concordant	Heterozygous	DRB1*03:01	DRB1*15:01	DRB1*03:01:01G/03:01:11/03:50/03:68N	DRB1*15:01:01G	96.7	100.0	45	49	100.0	100.0
316	Concordant	Heterozygous	DRB1*04:01	DRB1*07:01	DRB1*04:01:01/04:01:08	DRB1*07:01:01G	98.3	103.3	42	47	100.0	100.0

317	Concordant	Heterozygous	DRB1*04:04	DRB1*07:01	DRB1*04:04:01/04:23	DRB1*07:01:01G	103.3	96.7	48	39	100.0	100.0
318	Concordant	Heterozygous	DRB1*03:01	DRB1*13:02	DRB1*03:01:01G/03:01:11/03:50/03:68N	DRB1*13:02:01/13:109/13:128	103.3	63.3	58	32	100.0	100.0
319	Concordant	Heterozygous	DRB1*11:04	DRB1*15:01	DRB1*11:04:01/11:04:02/11:04:06	DRB1*15:01:01G	28.3	100.0	31	72	100.0	100.0
320	Concordant	Heterozygous	DRB1*01:03	DRB1*16:01	DRB1*01:03	DRB1*16:01:01	100.0	95.0	45	44	100.0	100.0
321	Concordant	Heterozygous	DRB1*01:03	DRB1*16:01	DRB1*01:03	DRB1*16:01:01	103.3	100.0	44	45	100.0	100.0
322	Concordant	Heterozygous	DRB1*03:01	DRB1*14:01	DRB1*03:01:01G/03:01:11/03:50/03:68N	DRB1*14:01:01G/14:113/14:114	100.0	96.7	44	48	100.0	100.0
323	Concordant	Heterozygous	DRB1*08:01	DRB1*11:01	DRB1*08:01:01G/08:01:05/08:39	DRB1*11:01:01G/11:01:02/11:01:06/11:100	100.0	53.3	70	31	100.0	100.0
324	Concordant	Heterozygous	DRB1*07:01	DRB1*14:01	DRB1*07:01:01G	DRB1*14:01:01G/14:113/14:114	98.3	100.0	45	42	100.0	100.0
325	Concordant	Heterozygous	DRB1*03:01	DRB1*11:01	DRB1*03:01:01G/03:01:11/03:50/03:68N	DRB1*11:01:01G/11:01:02/11:01:06/11:100	106.7	70.0	62	31	100.0	100.0
326	Concordant	Heterozygous	DRB1*11:03	DRB1*15:01	DRB1*11:03	DRB1*15:01:01G	96.7	98.3	41	53	100.0	100.0
327	Concordant	Heterozygous	DRB1*08:01	DRB1*09:01	DRB1*08:01:01G/08:01:05/08:39	DRB1*09:01:02/09:09	100.0	96.7	45	47	100.0	100.0
329	Concordant	Heterozygous	DRB1*04:01	DRB1*04:04	DRB1*04:01:01/04:01:08	DRB1*04:04:01/04:23	96.7	98.3	43	51	100.0	100.0
331	Concordant	Heterozygous	DRB1*04:01	DRB1*15:01	DRB1*04:01:01/04:01:08	DRB1*15:01:01G	100.0	95.0	55	39	100.0	100.0
332	Concordant	Heterozygous	DRB1*01:01	DRB1*15:01	DRB1*01:01:01/01:01:05	DRB1*15:01:01G	101.7	100.0	44	45	100.0	100.0
333	Concordant	Heterozygous	DRB1*04:01	DRB1*15:01	DRB1*04:01:01/04:01:08	DRB1*15:01:01G	98.3	98.3	45	48	100.0	100.0
334	Concordant	Heterozygous	DRB1*01:01	DRB1*15:01	DRB1*01:01:01/01:01:05	DRB1*15:01:01G	101.7	98.3	50	40	100.0	100.0
335	Concordant	Heterozygous	DRB1*13:01	DRB1*13:02	DRB1*13:01:01G/13:01:08/13:105/13:112	DRB1*13:02:01/13:109/13:128	61.0	39.0	94	94	100.0	100.0
336	Concordant	Heterozygous	DRB1*07:01	DRB1*11:04	DRB1*07:01:01G	DRB1*11:04:01/11:04:02/11:04:06	105.0	58.3	65	31	100.0	100.0
338	Concordant	Heterozygous	DRB1*01:03	DRB1*04:01	DRB1*01:03	DRB1*04:01:01/04:01:08	100.0	98.3	49	42	100.0	100.0
339	Concordant	Heterozygous	DRB1*01:02	DRB1*15:01	DRB1*01:02:01	DRB1*15:01:01G	100.0	98.3	53	38	100.0	100.0
340	Concordant	Heterozygous	DRB1*01:01	DRB1*11:01	DRB1*01:01:01/01:01:05	DRB1*11:01:01G/11:01:02/11:01:06/11:100	101.7	90.0	55	39	100.0	100.0
341	Concordant	Heterozygous	DRB1*07:01	DRB1*14:01	DRB1*07:01:01G	DRB1*14:01:01G/14:113/14:114	101.7	96.7	46	44	100.0	100.0
342	Concordant	Heterozygous	DRB1*04:04	DRB1*13:01	DRB1*04:04:01/04:23	DRB1*13:01:01G/13:01:08/13:105/13:112	100.0	18.3	76	27	100.0	100.0
343	Concordant	Heterozygous	DRB1*08:01	DRB1*15:01	DRB1*08:01:01G/08:01:05/08:39	DRB1*15:01:01G	98.3	98.3	42	51	100.0	100.0
344	Concordant	Heterozygous	DRB1*01:02	DRB1*15:01	DRB1*01:02:01	DRB1*15:01:01G	101.7	95.0	48	42	100.0	100.0
345	Concordant	Heterozygous	DRB1*07:01	DRB1*11:01	DRB1*07:01:01G	DRB1*11:01:01G/11:01:02/11:01:06/11:100	100.0	86.7	59	36	100.0	100.0
346	Concordant	Heterozygous	DRB1*11:04	DRB1*13:01	DRB1*11:04:01/11:04:02/11:04:06	DRB1*13:01:01G/13:01:08/13:105/13:112	100.0	75.0	65	30	100.0	100.0
347	Concordant	Heterozygous	DRB1*04:01	DRB1*11:01	DRB1*04:01:01/04:01:08	DRB1*11:01:01G/11:01:02/11:01:06/11:100	100.0	81.7	62	34	100.0	100.0
348	Concordant	Heterozygous	DRB1*03:01	DRB1*15:01	DRB1*03:01:01G/03:01:11/03:50/03:68N	DRB1*15:01:01G	93.3	100.0	39	55	100.0	100.0
349	Concordant	Heterozygous	DRB1*03:01	DRB1*04:01	DRB1*03:01:01G/03:01:11/03:50/03:68N	DRB1*04:01:01/04:01:08	100.0	100.0	42	50	100.0	100.0
350	Concordant	Heterozygous	DRB1*01:01	DRB1*13:01	DRB1*01:01:01/01:01:05	DRB1*13:01:01G/13:01:08/13:105/13:112	101.7	58.3	68	30	100.0	100.0
351	Concordant	Heterozygous	DRB1*11:01	DRB1*16:01	DRB1*11:01:01G/11:01:02/11:01:06/11:100	DRB1*16:01:01	86.7	100.0	38	59	100.0	100.0
352	Concordant	Heterozygous	DRB1*11:04	DRB1*15:01	DRB1*11:04:01/11:04:02/11:04:06	DRB1*15:01:01G	71.7	100.0	34	64	100.0	100.0
353	Concordant	Heterozygous	DRB1*03:01	DRB1*04:04	DRB1*03:01:01G/03:01:11/03:50/03:68N	DRB1*04:04:01/04:23	103.3	100.0	45	44	100.0	100.0
354	Concordant	Heterozygous	DRB1*01:01	DRB1*07:01	DRB1*01:01:01/01:01:05	DRB1*07:01:01G	108.3	98.3	50	37	100.0	100.0
355	Concordant	Heterozygous	DRB1*11:01	DRB1*11:04	DRB1*11:01:01G/11:01:02/11:01:06/11:100	DRB1*11:04:01/11:04:02/11:04:06	54.4	45.7	92	92	99.9	100.0
356	Concordant	Heterozygous	DRB1*03:01	DRB1*15:01	DRB1*03:01:01G/03:01:11/03:50/03:68N	DRB1*15:01:01G	96.7	100.0	41	53	100.0	100.0
357	Concordant	Heterozygous	DRB1*01:01	DRB1*15:01	DRB1*01:01:01/01:01:05	DRB1*15:01:01G	101.7	93.3	52	39	100.0	100.0
358	Concordant	Heterozygous	DRB1*04:07	DRB1*11:01	DRB1*04:07:01G/04:07:03	DRB1*11:01:01G/11:01:02/11:01:06/11:100	100.0	80.0	63	34	100.0	100.0
359	Concordant	Heterozygous	DRB1*04:02	DRB1*12:01	DRB1*04:02:01	DRB1*12:01:01G	108.3	90.0	49	40	100.0	100.0
361	Concordant	Heterozygous	DRB1*03:01	DRB1*04:01	DRB1*03:01:01G/03:01:11/03:50/03:68N	DRB1*04:01:01/04:01:08	100.0	100.0	44	49	100.0	100.0
362	Concordant	Heterozygous	DRB1*01:01	DRB1*13:03	DRB1*01:01:01/01:01:05	DRB1*13:03:01/13:03:02	98.3	98.3	48	46	100.0	100.0
363	Concordant	Heterozygous	DRB1*04:01	DRB1*15:01	DRB1*04:01:01/04:01:08	DRB1*15:01:01G	96.7	100.0	39	54	100.0	100.0
364	Concordant	Heterozygous	DRB1*07:01	DRB1*15:01	DRB1*07:01:01G	DRB1*15:01:01G	96.7	101.7	39	51	100.0	100.0
365	Concordant	Heterozygous	DRB1*12:01	DRB1*13:02	DRB1*12:01:01G	DRB1*13:02:01/13:109/13:128	103.3	93.3	54	37	100.0	100.0
366	Concordant	Heterozygous	DRB1*01:03	DRB1*07:01	DRB1*01:03	DRB1*07:01:01G	100.0	90.0	60	35	100.0	100.0
367	Concordant	Heterozygous	DRB1*04:04	DRB1*11:04	DRB1*04:04:01/04:23	DRB1*11:04:01/11:04:02/11:04:06	100.0	96.7	55	39	100.0	100.0
368	Concordant	Heterozygous	DRB1*11:03	DRB1*11:04	DRB1*11:03	DRB1*11:04:01/11:04:02/11:04:06	81.7	68.3	66	60	100.0	100.0
369	Concordant	Heterozygous	DRB1*14:01	DRB1*15:02	DRB1*14:01:01G/14:113/14:114	DRB1*15:02:01/15:19	100.0	98.3	49	45	100.0	100.0
370	Concordant	Heterozygous	DRB1*01:01	DRB1*03:01	DRB1*01:01:01/01:01:05	DRB1*03:01:01G/03:01:11/03:50/03:68N	98.3	106.7	45	40	100.0	100.0
371	Concordant	Heterozygous	DRB1*01:02	DRB1*15:01	DRB1*01:02:01	DRB1*15:01:01G	123.3	75.0	53	31	100.0	100.0
372	Concordant	Heterozygous	DRB1*03:01	DRB1*13:01	DRB1*03:01:01G/03:01:11/03:50/03:68N	DRB1*13:01:01G/13:01:08/13:105/13:112	101.7	23.3	76	27	100.0	100.0
373	Concordant	Heterozygous	DRB1*13:02	DRB1*15:01	DRB1*13:02:01/13:109/13:128	DRB1*15:01:01G	36.7	100.0	27	74	100.0	100.0
374	Concordant	Heterozygous	DRB1*01:02	DRB1*04:01	DRB1*01:02:01	DRB1*04:01:01/04:01:08	98.3	108.3	44	42	100.0	100.0
375	Concordant	Heterozygous	DRB1*01:03	DRB1*15:01	DRB1*01:03	DRB1*15:01:01G	96.7	100.0	38	56	100.0	100.0
376	Concordant	Heterozygous	DRB1*04:01	DRB1*13:03	DRB1*04:01:01/04:01:08	DRB1*13:03:01/13:03:02	100.0	98.3	51	44	100.0	100.0
377	Concordant	Heterozygous	DRB1*01:02	DRB1*03:01	DRB1*01:02:01	DRB1*03:01:01G/03:01:11/03:50/03:68N	105.0	106.7	42	40	100.0	100.0
378	Concordant	Heterozygous	DRB1*08:06	DRB1*15:01	DRB1*08:06	DRB1*15:01:01G	98.3	100.0	44	50	100.0	100.0
379	Concordant	Heterozygous	DRB1*07:01	DRB1*11:01	DRB1*07:01:01G	DRB1*11:01:01G/11:01:02/11:01:06/11:100	101.7	96.7	53	39	100.0	100.0
380	Concordant	Heterozygous	DRB1*12:01	DRB1*14:01	DRB1*12:01:01G	DRB1*14:01:01G/14:113/14:114	100.0	76.7	64	33	100.0	100.0
381	Concordant	Heterozygous	DRB1*07:01	DRB1*15:01	DRB1*07:01:01G	DRB1*15:01:01G	101.7	98.3	49	43	100.0	100.0
382	Concordant	Heterozygous	DRB1*12:01	DRB1*15:01	DRB1*12:01:01G	DRB1*15:01:01G	100.0	96.7	54	40	100.0	100.0
383	Concordant	Heterozygous	DRB1*08:01	DRB1*13:02	DRB1*08:01:01G/08:01:05/08:39	DRB1*13:02:01/13:109/13:128	100.0	78.3	64	33	100.0	100.0
21	Concordant	Homozygous	DRB1*15:01	DRB1*15:01	DRB1*15:01:01G	DRB1*15:01:01G	100.0	100.0	87	87	100.0	100.0
23	Concordant	Homozygous	DRB1*03:01	DRB1*03:01	DRB1*03:01:01G/03:01:11/03:50/03:68N	DRB1*03:01:01G/03:01:11/03:50/03:68N	100.0	100.0	95	95	100.0	100.0
26	Concordant	Homozygous	DRB1*11:04	DRB1*11:04	DRB1*11:04:01/11:04:02/11:04:06	DRB1*11:04:01/11:04:02/11:04:06	100.0	100.0	94	94	100.0	100.0
46	Concordant	Homozygous	DRB1*08:01	DRB1*08:01	DRB1*08:01:01G/08:01:05/08:39	DRB1*08:01:01G/08:01:05/08:39	100.0	100.0	95	95	100.0	100.0
54	Concordant	Homozygous	DRB1*04:01	DRB1*04:01	DRB1*04:01:01/04:01:08	DRB1*04:01:01/04:01:08	100.0	100.0	94	94	100.0	100.0
71	Concordant	Homozygous	DRB1*03:01	DRB1*03:01	DRB1*03:01:01G/03:01:11/03:50/03:68N	DRB1*03:01:01G/03:01:11/03:50/03:68N	100.0	100.0	91	91	100.0	100.0
73	Concordant	Homozygous	DRB1*01:01	DRB1*01:01	DRB1*01:01:01/01:01:05	DRB1*01:01:01/01:01:05	100.0	100.0	94	94	100.0	100.0
102	Concordant	Homozygous	DRB1*14:01	DRB1*14:01	DRB1*14:01:01G/14:113/14:114	DRB1*14:01:01G/14:113/14:114	100.0	100.0	94	94	100.0	100.0
111	Concordant	Homozygous	DRB1*03:01	DRB1*03:01	DRB1*03:01:01G/03:01:11/03:50/03:68N	DRB1*03:01:01G/03:01:11/03:50/03:68N	100.0	100.0	90	90	100.0	100.0
120	Concordant	Homozygous	DRB1*07:01	DRB1*07:01	DRB1*07:01:01G	DRB1*07:01:01G	100.0	100.0	94	94	100.0	100.0

122	Concordant	Homozygous	DRB1*03:01	DRB1*03:01	DRB1*03:01:01G/03:01:11/03:50/03:68N	DRB1*03:01:01G/03:01:11/03:50/03:68N	100.0	100.0	93	93	100.0	100.0
141	Concordant	Homozygous	DRB1*03:01	DRB1*03:01	DRB1*03:01:01G/03:01:11/03:50/03:68N	DRB1*03:01:01G/03:01:11/03:50/03:68N	100.0	100.0	93	93	100.0	100.0
142	Concordant	Homozygous	DRB1*01:01	DRB1*01:01	DRB1*01:01:01/01:01:05	DRB1*01:01:01/01:01:05	100.0	100.0	95	95	100.0	100.0
175	Concordant	Homozygous	DRB1*15:01	DRB1*15:01	DRB1*15:01:01G	DRB1*15:01:01G	100.0	100.0	88	88	100.0	100.0
181	Concordant	Homozygous	DRB1*07:01	DRB1*07:01	DRB1*07:01:01G	DRB1*07:01:01G	100.0	100.0	95	95	100.0	100.0
185	Concordant	Homozygous	DRB1*11:01	DRB1*11:01	DRB1*11:01:01G/11:01:02/11:01:06/11:100	DRB1*11:01:01G/11:01:02/11:01:06/11:100	100.0	100.0	92	92	100.0	100.0
205	Concordant	Homozygous	DRB1*15:01	DRB1*15:01	DRB1*15:01:01G	DRB1*15:01:01G	100.0	100.0	84	84	100.0	100.0
230	Concordant	Homozygous	DRB1*15:01	DRB1*15:01	DRB1*15:01:01G	DRB1*15:01:01G	100.0	100.0	86	86	100.0	100.0
247	Concordant	Homozygous	DRB1*07:01	DRB1*07:01	DRB1*07:01:01G	DRB1*07:01:01G	100.0	100.0	95	95	100.0	100.0
255	Concordant	Homozygous	DRB1*01:01	DRB1*01:01	DRB1*01:01:01/01:01:05	DRB1*01:01:01/01:01:05	112.5	112.5	84	84	100.0	100.0
256	Concordant	Homozygous	DRB1*04:01	DRB1*04:01	DRB1*04:01:01/04:01:08	DRB1*04:01:01/04:01:08	100.0	100.0	95	95	100.0	100.0
266	Concordant	Homozygous	DRB1*11:04	DRB1*11:04	DRB1*11:04:01/11:04:02/11:04:06	DRB1*11:04:01/11:04:02/11:04:06	100.0	100.0	95	95	100.0	100.0
271	Concordant	Homozygous	DRB1*03:01	DRB1*03:01	DRB1*03:01:01G/03:01:11/03:50/03:68N	DRB1*03:01:01G/03:01:11/03:50/03:68N	100.0	100.0	90	90	100.0	100.0
283	Concordant	Homozygous	DRB1*12:01	DRB1*12:01	DRB1*12:01:01G	DRB1*12:01:01G	100.0	100.0	95	95	100.0	100.0
287	Concordant	Homozygous	DRB1*11:01	DRB1*11:01	DRB1*11:01:01G/11:01:02/11:01:06/11:100	DRB1*11:01:01G/11:01:02/11:01:06/11:100	100.0	100.0	95	95	100.0	100.0
298	Concordant	Homozygous	DRB1*07:01	DRB1*07:01	DRB1*07:01:01G	DRB1*07:01:01G	100.0	100.0	95	95	100.0	100.0
300	Concordant	Homozygous	DRB1*03:01	DRB1*03:01	DRB1*03:01:01G/03:01:11/03:50/03:68N	DRB1*03:01:01G/03:01:11/03:50/03:68N	100.0	100.0	92	92	100.0	100.0
302	Concordant	Homozygous	DRB1*15:01	DRB1*15:01	DRB1*15:01:01G	DRB1*15:01:01G	100.0	100.0	87	87	100.0	100.0
305	Concordant	Homozygous	DRB1*07:01	DRB1*07:01	DRB1*07:01:01G	DRB1*07:01:01G	100.0	100.0	94	94	100.0	100.0
328	Concordant	Homozygous	DRB1*03:01	DRB1*03:01	DRB1*03:01:01G/03:01:11/03:50/03:68N	DRB1*03:01:01G/03:01:11/03:50/03:68N	100.0	100.0	92	92	100.0	100.0
330	Concordant	Homozygous	DRB1*03:01	DRB1*03:01	DRB1*03:01:01G/03:01:11/03:50/03:68N	DRB1*03:01:01G/03:01:11/03:50/03:68N	100.0	100.0	91	91	100.0	100.0
